# A cross-sectional exploration of the dietary inflammation index association with cardiovascular disease in gout: application of machine learning algorithms

**DOI:** 10.3389/fnut.2025.1591472

**Published:** 2025-09-18

**Authors:** Qiang Zhang, Xue-bing Lyu, Chang-quan Liu, Wei-zhen Zhang, Yu-guang Wang, Wei-zhe Deng, Xuan-hua Yu

**Affiliations:** ^1^Department of Rheumatology and Chinese Medicine, The 962nd Hospital of the Chinese PLA, Harbin, China; ^2^Department of Rheumatology, People's Hospital Affiliated to Fujian University of Traditional Chinese Medicine, Fuzhou, China

**Keywords:** gout, dietary inflammation index, hyperuricemia, cardiovascular disease, machine learning

## Abstract

**Objective:**

Gout is a condition strongly associated with dietary patterns and elevated risk of cardiovascular disease (CVD) in affected individuals. Given the potential influence of dietary diversity on inflammatory responses, this study aimed to explore the association between the dietary inflammatory index (DII) and CVD prevalence in gout patients.

**Methods:**

Data from gout patients in NHANES 2007–2018 were extracted for analysis. Correlation matrices were employed to examine the relationships among 28 dietary inflammation indices. Machine learning algorithms were utilized to identify key features for constructing a covariate subset for the final model, and Random Forest SHAP interpretations were applied to assess variable risk factors. The relationship between DII and CVD risk in gout patients was assessed using multi-model logistic regression. RCS were applied to evaluate the risk trend and to assess model discrimination, predictive probability, and clinical benefit using ROC, calibration curves, and DCA, respectively. Subgroup analysis was evaluated the heterogeneity in CVD across different populations.

**Results:**

1,437 gout patients met inclusion criteria were included in the study, with mean age of 60.84 years, consisting of 435 females (31.23%) and 1,002 males (68.77%), and an overall CVD prevalence of 32.92%. DII was linearly associated with CVD risk (*P* for overall = 0.002; *P* for nonlinear = 0.810). In the final model, DII was positively associated with CVD risk, showing 118% increased risk in Q4 compared to Q1 (OR: 2.18, 95%CI: 1.52–3.13, *p* < 0.001). The constructed model exhibited stability performance (AUC = 0.750, 95%CI: 0.722–0.775). Segmented subgroup analysis indicated that gout patients with high DII (> 1.934) had a increased risk of CVD (OR: 1.33, 95%CI: 0.06–1.65, *p* = 0.012), while those younger than 60 years had higher risk (OR: 2.19, 95%CI: 1.36–3.54, *p* = 0.001).

**Conclusion:**

Higher DII was associated with increased prevalence of CVD in gout patients. Dietary modification may serve as an effective strategy for preventing disease progression and reducing CVD risk. Our findings support the clinical development of dietary and nutritional guidance programs.

## Introduction

Gout is a group of disorders resulting from purine metabolism disturbances, as both metabolic and rheumatic diseases characterized by prolonged hyperuricemia and acute, self-limiting arthritic flare-ups as the primary clinical manifestations (1, 2). As the disease progresses, urate crystals continue to accumulate in the periarticular and subcutaneous tissues, as well as in the kidneys, leading to the formation of gout stones. In some cases, bone erosion and renal failure, advancing to refractory gout, which significantly impairs patients’ functional capacity and quality of life (3). From 1990 to 2019, the global number of gout patients increased from 22 million to 53 million. The age-standardized prevalence rate rose from 532.99 to 652.24 per 10,000 individuals, with the male-to-female prevalence ratio remaining at 3:1. The incidence of gout grew by 70.15% in male and 68.70% in female (4). A meta-analysis using Global Burden of Disease (GBD) data found that the all-cause treatment costs for employed, elderly, and refractory gout populations were $4,733, $16,925, and $18,362, respectively. These costs were positively correlated with blood uric acid levels and the frequency of acute gout episodes (5).

Previous epidemiological studies have shown that gout is associated with elevated risk of cardiovascular diseases (CVD), including coronary heart disease (CHD), myocardial infarction (MI), peripheral artery disease (PAD), congestive heart failure (CHF), and CVD mortality (6, 7). A large epidemiological study in Asia reported a 57% increase in the overall risk of CVD in patients with gout compared to the general population (8). The 2020 American College of Rheumatology (ACR) guidelines for gout management recommend screening for CVD comorbidities and updating the management of gout patients (9). Managing CVD complications in gout patients has become a significant public health challenge.

Gout is strongly associated with the consumption of rich foods and uncontrolled alcohol intake, while chronic low-grade inflammation from these dietary factors contributes to elevated blood uric acid levels and frequent gout flare-ups (10, 11). Diets such as the DASH diet, Mediterranean diet, and those rich in fiber and vegetables have been shown to reduce inflammation, which in turn lowers the prevalence of CVD (12, 13). Dietary inflammation may contribute significantly to various diseases, but fewer studies have explored how modifying dietary patterns can reduce the risk of CVD in gout patients. The dietary inflammation index (DII) is an innovative dietary tool designed to directly assess the inflammatory potential of the overall diet (14, 15). The DII evaluates the inflammatory potential of the diet based on 45 components and has been validated through various serum markers of inflammation, such as C-reactive protein (CRP) and interleukins (IL) (16). A review of the literature suggests that DII plays a critical role in regulating inflammation in metabolic diseases, neuropsychiatric disorders, respiratory conditions, and malignancy-associated chronic diseases (17–20). However, No clinical studies on the relationship between the DII and the risk of CVD in gout participants were found through searching the PubMed and Web of Science databases, and the extent to which DII influences the progression of CVD in this population remains unclear.

Highly representative sample was selected through a rigorous screening process using data from the National Health and Nutrition Examination Survey (NHANES) 2007–2018. Machine learning algorithms were employed to construct models for exploring and analyzing the potential association between DII and CVD risk in gout. This study aims to assist clinicians in more accurately assessing CVD risk in gout patients, identifying those at high risk, and providing a foundation for effective disease management strategies.

### Study population

Data from the NHANES, a multi-institutional series designed to assess the health and nutritional status of both adults and children in the U.S. The survey protocol was approved by the Institutional Review Board (IRB) of the National Center for Health Statistics (NCHS). All participants provided written informed consent, ensuring the study’s ethical compliance. Data for this study were obtained from the official NHANES website, ensuring data transparency and accessibility.

**Figure 1:**
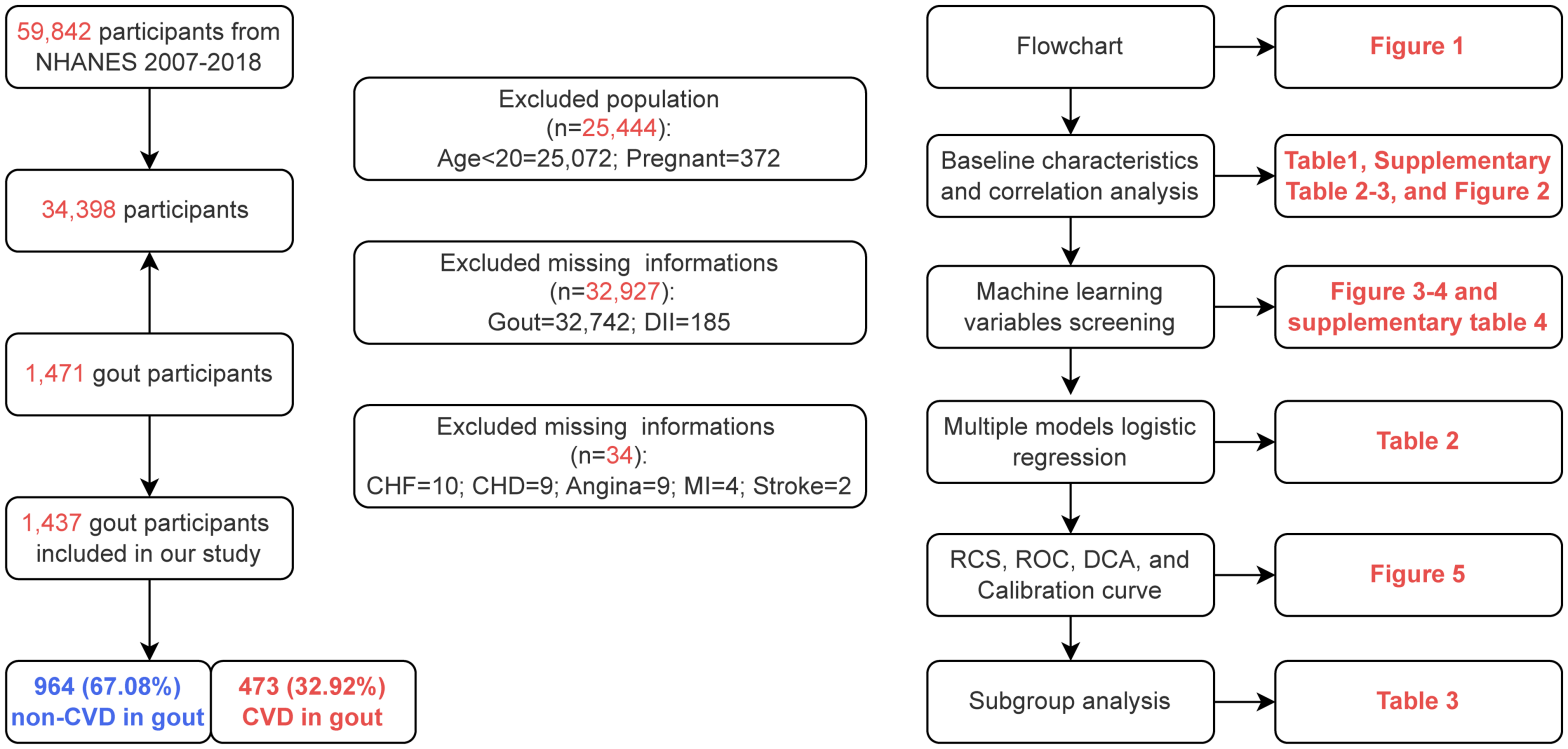
Flowchart of participant screening and technology roadmap.

Data from 6 NHANES cycles conducted between 2007 to 2018 were included in this study, which initially screened 59,842 participants. Strict exclusion criteria were applied to maintain the rigor of the study design. A total of 44,562 ineligible participants were excluded, including those younger than 20 years (*n* = 25,072), pregnant female (*n* = 372), participants missing gout information (*n* = 32,740), missing CVD information (*n* = 34), and missing DII information (*n* = 185). After applying these exclusions, 1,437 eligible participants remained in the study. Figure 1 illustrates the participant screening process.

### Definition of gout

The diagnosis of gout was based on a self-reported physician’s diagnosis, with participants being asked, “doctor ever told you that you have gout?” If a participant answered “yes,” they were considered to have the condition (21).

### Definition of cardiovascular disease

The diagnosis of CVD was established through self-reported physician diagnoses obtained via an individual interview using a standardized medical condition questionnaire. CVD was defined as self-reported physician diagnoses of CHF, CHD, angina, MI, or stroke. If a participant answered “yes,” they were considered to have the condition (22).

### Definition of dietary inflammation index

45 specific foods and nutrients were associated with various inflammatory or anti-inflammatory biomarkers, and the inflammatory potential of each dietary component was scored based on biomarkers, including CRP, tumor necrosis factor (TNF)-*α*, IL-1β, IL-4, IL-6, and IL-10. A score of +1 was assigned to dietary components that significantly increased inflammatory biomarkers, and a score of −1 was assigned to those that decreased them (15, 16). The global means and standard deviations of 45 food parameters were calculated using data from 11 countries (23, 24).

The NHANES collected dietary information via 24 h dietary recall interviews conducted at mobile examination center (MEC). Two 24 h dietary recall interviews were used to calculate DII for each participant. However, due to missing nutrient data in the NHANES dietary database (25), 28 food parameters were included in this cross-sectional study to calculate DII. The parameters analyzed included energy, protein, carbohydrates, dietary fiber, total fat, saturated fat, monounsaturated fatty acids, polyunsaturated fatty acids, cholesterol, vitamin (Vit) A, *β*-carotene, thiamine, riboflavin, niacin, VitB6, VitB12, VitC, VitD, VitE, magnesium, iron, zinc, selenium, caffeine, alcohol, and n-3 and n-6 fatty acids.

### Covariates in NHANES

Covariates for this cross-sectional study included demographic data from NHANES 2007–2018, physical examination results, laboratory examinations, and questionnaire data. These covariates included information on age, gender, body mass index (BMI), blood pressure, smoke, drink, sleep disorder, 12 self-reported comorbidities, and 21 laboratory examinations. Drink status was defined as “had drink more than 12 times in the past year” or “drink more than once a month,” and smoke status as “had smoke more than 100 cigarettes in lifetime” or “current smoke.” Hypertension was defined as “systolic blood pressure average (SBP Avg) ≥ 135 mmHg, diastolic blood pressure average (DBP Avg) ≥ 85 mmHg, ever been told by a doctor or other health professional that had hypertension, or taking prescription for hypertension”.

According to World Health Organization (WHO) criteria, participants with fasting blood glucose ≥ 126 mg/dL, 2 h blood glucose ≥ 200 mg/dL on the oral glucose tolerance test (OGTT), or glycosylated hemoglobin (HbA1c) ≥ 6.5% were defined as having diabetes (26). Chronic kidney disease (CKD) was defined according to KDIGO criteria: eGFR < 60 mL/min; or a total urine protein (UPRO) /creatinine ratio > 30 mg/g when eGFR ≥ 60 mL/min. Participants met one of these criteria were defined as CKD (27). Asthma, chronic obstructive pulmonary disease (COPD), kidney stones, and cancer were assessed based on self-reported health data with the question, “Doctor told you had asthma, COPD, kidney stones, cancer, or malignancy.” Blood and urine specimens were collected at the MEC and sent to a standardized laboratory for testing.

### Statistical analysis

The baseline study population across 6 cycles was weighted according to the statistical methods recommended by NHANES analysis guidelines. Missing data were imputed using simple interpolation, with no more than 20% of model variables missing. In the descriptive analysis, continuous variables were expressed as means with standard error (SE), while categorical variables were presented as frequencies. The Chi-square test was used for categorical data, and the t-test was applied to continuous variables for group comparison.

Correlation matrix was plotted to show the relationship among the 28 DIIs. Important features were selected using the Boruta and Random Forest algorithms to construct a subset of covariates for the final model, and risk measures of the assessment variables were interpreted using Random Forest SHAP. Gout patients were grouped according to DII quartiles for logistic regression analysis. RCS was applied to validate the risk trend, and discrimination, predictive probability, and clinical benefit were assessed using ROC, calibration curves, and DCA, respectively. Segmented subgroup analysis was performed to assess the heterogeneity of CVD occurrence across different populations.

Statistical analysis and data visualization were conducted using SPSS 27.0.1, R-studio 4.4.2 and DCPM 6.03.1. *p* value < 0.05 was considered statistically significant.

## Results

### Baseline characteristics of study population

The baseline characteristics of the study population, which included 1,437 gout participants with mean age of 60.84 years, 435 females (31.23%) and 1,002 males (68.77%) were presented in Table 1. The overall prevalence of CVD was 32.92%, with 191 (9.54%) cases of CHF, 192 (12.64%) cases of CHD, 118 (7.75%) cases of Angina, 204 (11.45%) cases of MI, and 148 (7.44%) cases of Stroke. CVD participants were older than those without CVD (67.24 vs. 58.49, *p* < 0.001). The prevalence of hypertension, diabetes, asthma, COPD, CKD, and kidney stones was also higher among CVD participants. The prevalence of kidney stones was also higher in participants with CVD.

**Table 1:** Basic characteristics of participants in gout.

Covariates	Total	Non-CVD	CVD	*P*-value
	*n* = 1,437	*n* = 964	*n* = 473	
DII, mean (SE)	1.60 (0.07)	1.46 (0.08)	1.96 (0.11)	<0.001
Age, mean (SE)	60.84 (0.48)	58.49 (0.55)	67.24 (0.61)	<0.001
Gender, *n*(%)				0.812
Male	1,002 (68.77)	672 (69.06)	330 (68.00)	
Female	435 (31.23)	292 (30.94)	143 (32.00)	
SBP Avg, Mean (SE)	130.63 (0.72)	130.62 (0.82)	130.65 (1.35)	0.985
DBP Avg, Mean (SE)	71.46 (0.60)	72.91 (0.68)	67.51 (0.84)	<0.001
BMI, mean (SE)	32.10 (0.33)	31.95 (0.36)	32.50 (0.60)	0.412
Drink, *n*(%)	823 (59.60)	562 (61.78)	261 (53.66)	0.036
Smoke, *n*(%)	832 (56.56)	524 (54.51)	308 (62.15)	0.054
Sleep disorder, *n*(%)	553 (42.63)	334 (40.26)	219 (49.07)	0.021
Complications, *n*(%)				
Hypertension	1,051 (68.58)	649 (64.35)	402 (80.10)	<0.001
Diabetes	604 (34.22)	349 (28.93)	255 (48.63)	<0.001
CHF	191 (9.54)	0 (0.00)	191 (35.53)	–
CHD	192 (12.64)	0 (0.00)	192 (47.10)	–
Angina	118 (7.75)	0 (0.00)	118 (28.87)	–
MI	204 (11.45)	0 (0.00)	204 (42.64)	–
Stroke	148 (7.44)	0 (0.00)	148 (27.71)	–
Asthma	259 (17.15)	153 (15.20)	106 (22.46)	0.009
COPD	77 (3.83)	37 (2.26)	40 (8.13)	<0.001
CKD	615 (33.93)	347 (28.96)	268 (47.47)	<0.001
Kidney stone	253 (19.74)	157 (18.66)	96 (22.70)	0.265
Cancer	290 (22.20)	165 (19.68)	125 (29.08)	0.005
Laboratory examinations, mean (SE)				
WBC, 10^3^/uL	7.52 (0.09)	7.37 (0.11)	7.94 (0.16)	0.004
PLT, 10^3^/uL	229.86 (2.53)	233.68 (2.85)	219.45 (4.96)	0.013
RBC, 10^6^/uL	4.68 (0.02)	4.71 (0.02)	4.57 (0.05)	0.007
Hb, g/dL	14.32 (0.08)	14.46 (0.08)	13.94 (0.17)	0.003
eGFR, ml/min	75.62 (0.70)	79.08 (0.89)	66.20 (1.23)	<0.001
UPRO, ug/mL	98.69 (12.14)	76.66 (11.58)	158.74 (31.96)	0.016
BUN, mg/dL	17.36 (0.25)	16.39 (0.28)	20.00 (0.48)	<0.001
UA, mg/dL	6.55 (0.07)	6.54 (0.08)	6.57 (0.11)	0.828
Scr, mg/dL	1.13 (0.03)	1.09 (0.04)	1.24 (0.04)	0.011
ALT, U/L	27.05 (0.60)	28.12 (0.80)	24.14 (0.91)	0.003
AST, U/L	27.19 (0.41)	27.42 (0.53)	26.57 (0.86)	0.447
ALB, g/dL	4.19 (0.01)	4.22 (0.02)	4.10 (0.02)	<0.001
GLB, g/dL	2.88 (0.01)	2.86 (0.02)	2.95 (0.03)	0.019
GLU, mg/dL	113.99 (1.89)	112.57 (2.35)	117.87 (2.46)	0.109
HbA1c, %	6.03 (0.05)	5.96 (0.06)	6.23 (0.07)	0.004
Na, mmol/L	139.31 (0.16)	139.26 (0.18)	139.45 (0.18)	0.336
K, mmol/L	4.09 (0.01)	4.05 (0.01)	4.19 (0.02)	<0.001
TC, mg/dL	187.69 (1.82)	194.28 (2.15)	169.75 (3.08)	<0.001
TG, mg/dL	165.00 (4.32)	168.41 (5.43)	155.70 (4.11)	0.043
LDL-C, mg/dL	108.25 (1.14)	112.97 (1.35)	95.41 (1.60)	<0.001
HDL-C, mg/dL	47.95 (0.76)	48.52 (0.94)	46.37 (0.87)	0.073

DII: dietary inflammatory index, CVD: cardiovascular disease, SBP Avg: average systolic blood pressure, DBP Avg: average diastolic blood pressure, BMI: body mass index, CHF: congestive heart failure, CHD: coronary heart disease, MI: myocardial infarction, WBC: white blood cell, PLT: platelet, RBC: red blood cell, Hb: hemoglobin, eGFR: estimated glomerular filtration rate, UPRO: urinary protein, BUN: blood urea nitrogen, UA: uric acid, Scr: serum creatinine, ALT: alanine aminotransferase, AST: aspartate aminotransferase, ALB: albumin, GLB: globulin, GLU: glucose, HbA1c: glycated hemoglobin, TC: total cholesterol, TG: triglyceride, LDL-C: low density lipoprotein cholesterol, HDL-C: high density lipoprotein cholesterol. *P* value less than 0.05 indicates a significant difference.

21 laboratory examinations were included in the study. CVD participants exhibited higher DII (1.96 vs. 1.46, *p* < 0.001), UPRO, Scr, BUN, GLB, GLU, HbA1c, and K, and lower PLT, RBC, Hb, ALB, eGFR, ALT, TC, TG, and LDL compared to participants without CVD. The CVD prevalence of the Quartitles of DII were presented in Supplementary Table 2. Relative Q4 compared to Q1 showed a higher prevalence of CVD (34.56 vs. 18.69%, *p* < 0.001), CHF (15.10 vs. 4.87%, *p* < 0.001), and stroke (11.59 vs. 3.15%, *p* = 0.005).

### Correlation analysis of 28 dietary inflammation indices

**Figure 2:**
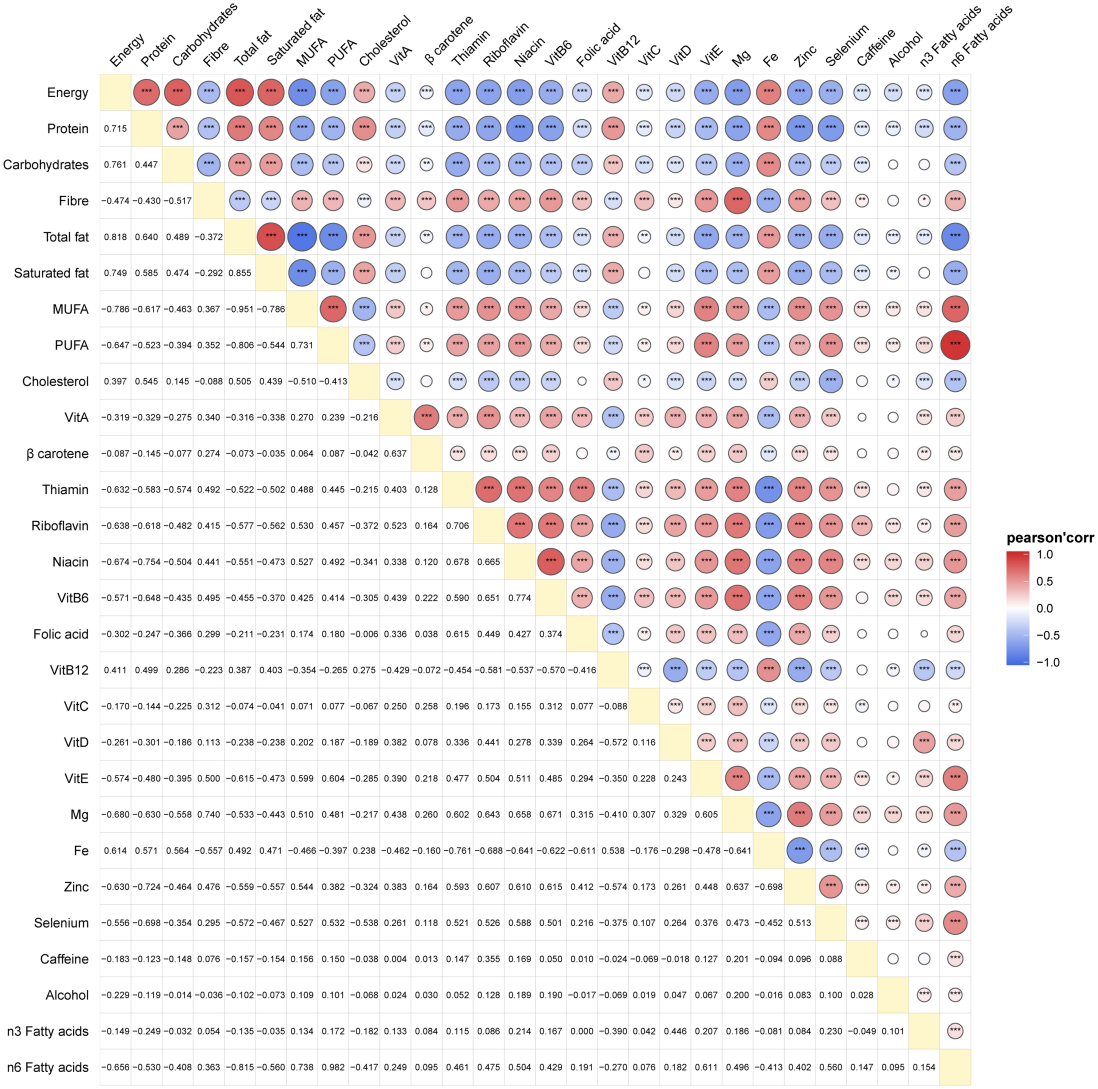
Relevant matrixof the 28 dietary inflammation indices of participants in gout.

The correlation matrix and coefficients for the 28 dietary inflammation indices are presented in Figure 2 and Supplementary Table 3. In the analysis of the 28 dietary inflammation indices in 1,437 gout patients, we found that energy, protein, carbohydrates, total fat, saturated fat, cholesterol, VitB12, and iron were associated with pro-inflammatory diets. In contrast, dietary fiber, monounsaturated fatty acids, polyunsaturated fatty acids, VitA, *β*-carotene, thiamine, riboflavin, niacin, VitB6, folic acid, VitC, VitD, VitE, magnesium, zinc, selenium, caffeine, alcohol, n-3 and n-6 fatty acids showed negative correlations of varying strengths.

### Feature selection of dietary inflammation indices with cardiovascular disease

**Figure 3:**
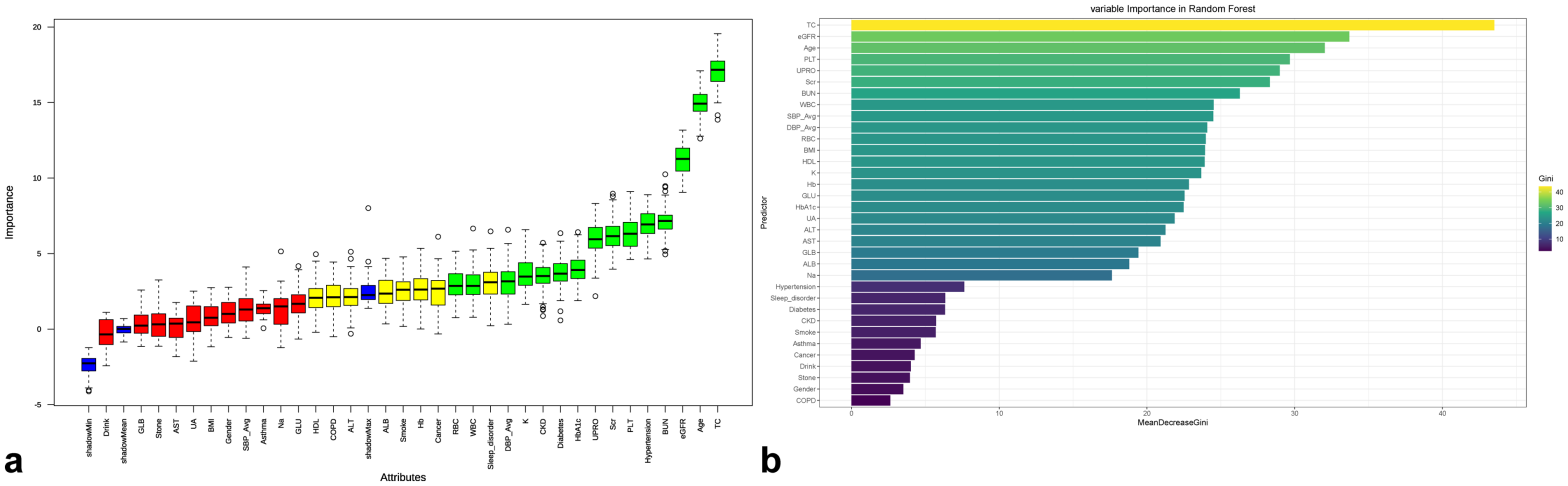
Ranking of variable importance based on Boruta **(a)** and Random Forest **(b)** algorithms of CVD in gout.

3 logistic regression models were constructed in our study to explore the association between DII and the prevalence of CVD. The adjusted covariates in Model 3 were selected through Boruta and Random Forest algorithm (Figure 3; Supplementary Figures 1–5; Supplementary Table 4). The screening covariates were as follows:

CVD: Age, Hypertension, Diabetes, BMI, eGFR, BUN, Scr, HbA1c, PLT, UPRO, WBC, SBP Avg, TC
CHF: Age, eGFR, BUN, Scr, UPRO, PLT, HbA1c, COPD, GLU, CKD, K, TC, SBP Avg
CHD: Age, eGFR, BUN, Scr, TC, Hb, UPRO, HDL-C, CKD, ALT, RBC, GLU, K, PLT
Angina: Age, CKD, BMI, eGFR, BUN, Scr, HbA1c, Hb, RBC, UPRO, DBP Avg, WBC, TC, PLT, HDL-C, SBP Avg
MI: Age, CKD, eGFR, BUN, Scr, TC, Hb, RBC, PLT, HDL-C, UPRO, DBP Avg, SBP Avg
Stroke: Age, CKD, eGFR, BUN, Scr, RBC, UPRO, ALT, Hb, PLT, GLU, TC, SBP Avg

### Multi-model analysis of cardiovascular disease and subtypes

**Figure 4:**
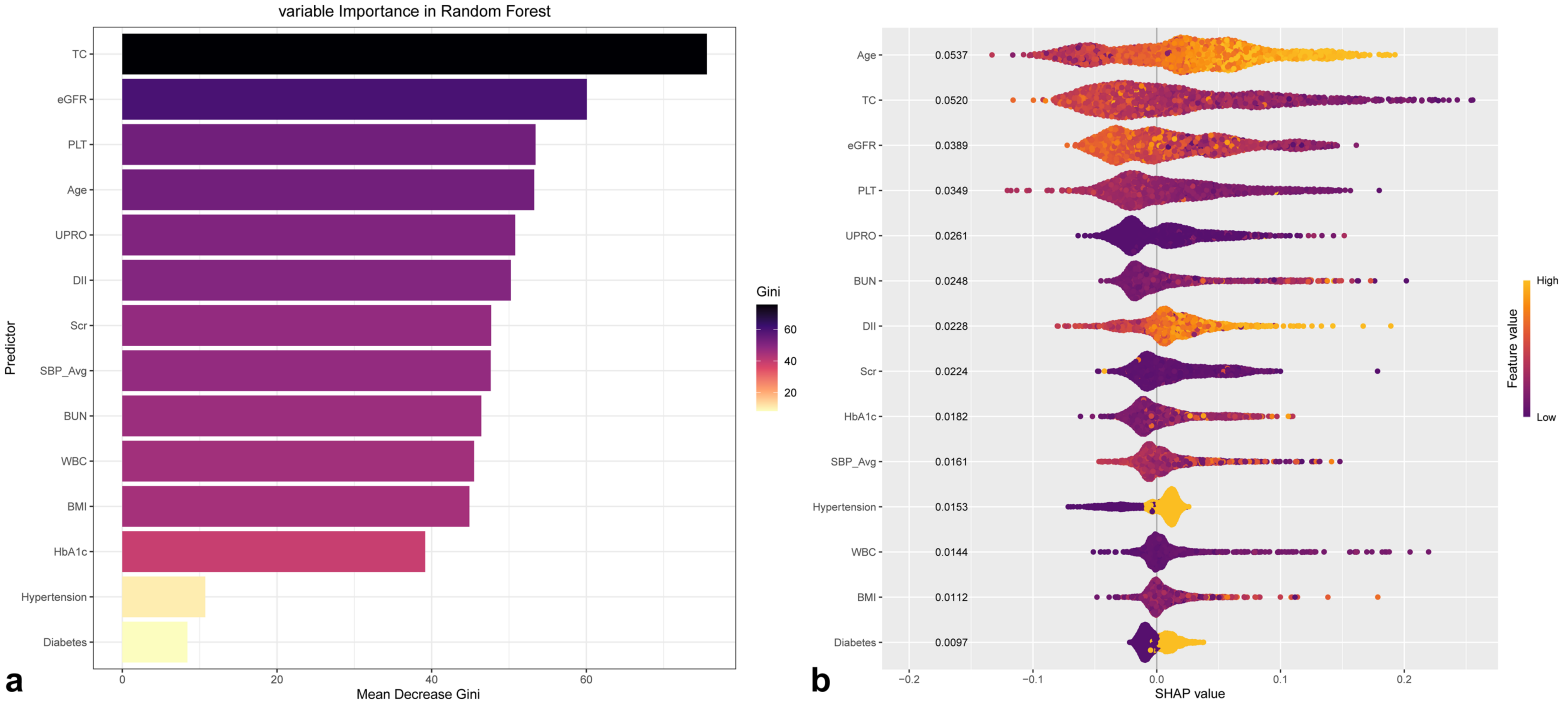
Distribution diagram of feature and SHAP values based on Random Forest of CVD in gout.

The results of univariate and multivariate logistic regression analyses for CVD and its four subtypes were presented in Table 2. Model 1 represents an unadjusted analysis, Model 2 is adjusted for sex and age, and Model 3 is adjusted for a subset of variables selected by eigenvalues. Random Forest variable importance ranking and SHAP values are also used to explain the contribution of each variable to the model outcomes, with the influence of the eigenvalues illustrated in Figure 4.

**Table 2:** Association between with CVD in gout.

Characteristic	DII OR (95%CI)				*P* for trend
	Q1	Q2	Q3	Q4	
CVD					
Model 1	Reference	2.26 (1.62, 3.14)*	1.75 (1.25, 2.44)*	2.41 (1.74, 3.35)*	< 0.001
Model 2	Reference	2.10 (1.49, 2.96)*	1.65 (1.16, 2.33)*	2.18 (1.55, 3.07)*	< 0.001
Model 3	Reference	1.94 (1.36, 2.78)*	1.56 (1.08, 2.25)*	2.18 (1.52, 3.13)*	< 0.001
CHF					
Model 1	Reference	2.12 (1.29, 3.49)*	2.02 (1.23, 3.34)*	2.82 (1.74, 4.57)*	< 0.001
Model 2	Reference	2.03 (1.23, 3.34)*	1.97 (1.19, 3.25)*	2.65 (1.63, 4.31)*	< 0.001
Model 3	Reference	1.89 (1.12, 3.19)*	1.68 (0.99, 2.85)	2.62 (1.57, 4.38)*	0.031
CHD					
Model 1	Reference	1.91 (1.23, 2.96)*	1.09 (0.68, 1.76)	1.58 (1.01, 2.48)*	0.305
Model 2	Reference	1.81 (1.16, 2.83)*	1.04 (0.64, 1.69)	1.45 (0.92, 2.29)	0.520
Model 3	Reference	1.70 (1.07, 2.69)*	0.98 (0.59, 1.62)	1.56 (0.96, 2.52)	0.361
Angina					
Model 1	Reference	1.58 (0.91, 2.73)	1.09 (0.61, 1.97)	1.57 (0.91, 2.72)	0.269
Model 2	Reference	1.41 (0.81, 2.45)	1.00 (0.55, 1.80)	1.37 (0.78, 2.38)	0.531
Model 3	Reference	1.31 (0.75, 2.31)	0.96 (0.52, 1.75)	1.32 (0.74, 2.36)	0.576
MI					
Model 1	Reference	2.02 (1.29, 3.18)*	1.52 (0.95, 2.44)*	2.06 (1.31, 3.23)*	0.013
Model 2	Reference	1.93 (1.22, 3.05)*	1.47 (0.92, 2.37)	1.92 (1.21, 3.02)*	0.031
Model 3	Reference	1.72 (1.08, 2.74)*	1.40 (0.86, 2.29)	1.91 (1.18, 3.08)*	0.030
Stroke					
Model 1	Reference	3.18 (1.70, 5.94)*	2.58 (1.36, 4.89)*	4.83 (2.64, 8.83)*	< 0.001
Model 2	Reference	3.05 (1.63, 5.71)*	2.50 (1.32, 4.76)*	4.57 (2.50, 8.38)*	< 0.001
Model 3	Reference	2.82 (1.49, 5.31)*	2.26 (1.18, 4.33)*	3.99 (2.15, 7.40)*	< 0.001

Model 1: unadjusted. Model 2: adjusted for gender, age. Model 3: CVD: Age, Hypertension, Diabetes, BMI, eGFR, BUN, Scr, HbA1c, PLT, UPRO, WBC, SBP Avg, TC; CHF: Age, eGFR, BUN, Scr, UPRO, PLT, HbA1c, COPD, GLU, CKD, K, TC, SBP Avg; CHD: Age, eGFR, BUN, Scr, TC, Hb, UPRO, HDL-C, CKD, ALT, RBC, GLU, K, PLT; Angina: Age, CKD, BMI, eGFR, BUN, Scr, HbA1c, Hb, RBC, UPRO, DBP Avg, WBC, TC, PLT, HDL-C, SBP Avg; MI: Age, CKD, eGFR, BUN, Scr, TC, Hb, RBC, PLT, HDL-C, UPRO, DBP Avg, SBP Avg; Stroke: Age, CKD, eGFR, BUN, Scr, RBC, UPRO, ALT, Hb, PLT, GLU, TC, SBP Avg. CVD: cardiovascular disease; CHF: congestive heart failure; CHD: coronary heart disease; MI: myocardial infarction. * or *P* value < 0.05 indicates statistical significance.

As the DII increased from Q1 to Q4, we observed a positive correlation between higher DII scores and a higher risk of CVD, CHF, CHD, MI, and stroke in both Model 1 and Model 2. In Model 2 and Model 3, higher DII was significantly associated with increased risk of CVD, CHF, MI, and stroke.

**Table 3:** Segmented subgroup analysis in gout.

Covariates	*n* (%)	DII		OR (95%CI)		*P*	*P* for interaction
		≤ 1.934	> 1.934	≤ 1.934	> 1.934		
Total	1,437 (100.00)	214/718	259/719	1	1.33 (1.06–1.65)	0.012	
Age							0.012
≤ 60	466 (32.43)	33/250	54/216	1	2.19 (1.36–3.54)	0.001	
> 60	971 (67.57)	181/468	205/503	1	1.09 (0.84–1.41)	0.508	
Gender							0.658
Male	1,002 (69.73)	173/569	157/433	1	1.30 (1.00–1.70)	0.051	
Female	435 (30.27)	41/149	102/286	1	1.46 (0.95–2.25)	0.087	
Hypertension							0.305
No	386 (26.86)	39/212	32/174	1	1.00 (0.60–1.68)	0.999	
Yes	1,051 (73.14)	175/506	227/545	1	1.35 (1.05–1.73)	0.019	
Diabetes							0.635
No	833 (57.97)	102/436	116/397	1	1.35 (0.99–1.84)	0.057	
Yes	604 (42.03)	112/282	143/322	1	1.21 (0.88–1.68)	0.244	
CKD							0.865
No	822 (57.20)	105/449	100/373	1	1.20 (0.87–1.65)	0.259	
Yes	615 (42.80)	109/269	159/346	1	1.25 (0.90–1.72)	0.178	

DII: dietary inflammatory index, OR: odds ratio, CI: confidence interval, CKD: chronic kidney disease. *P* value or *P* for interaction less than 0.05 for OR indicates a significant difference.

**Figure 5:**
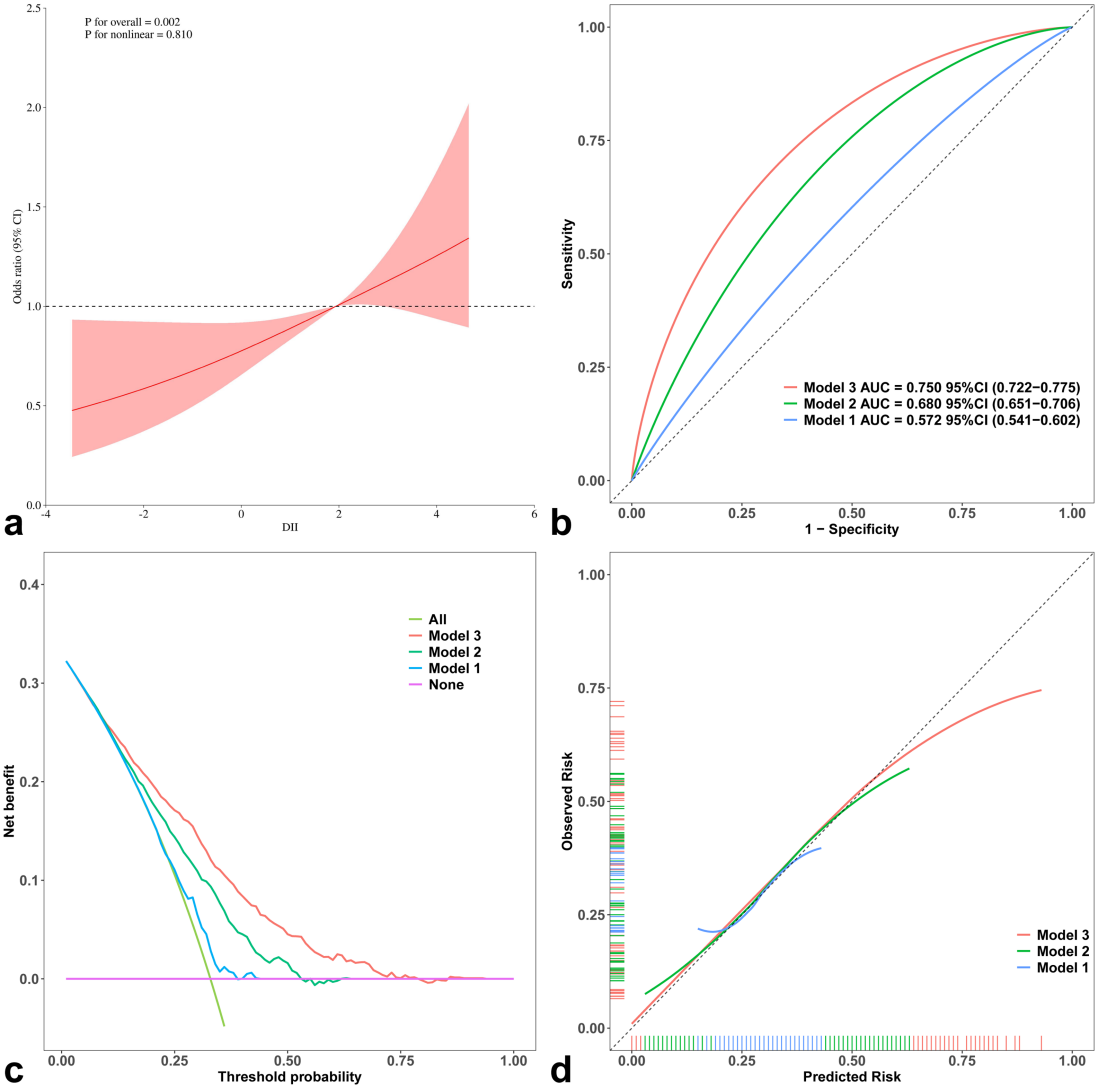
Performance evaluation of multi-model incorporating confounders with RCS **(a)**, ROC **(b)**, DCA **(c)** and calibration curve **(d)** of CVD in gout.

Figure 5 shows that DII was linearly associated with the risk of CVD in gout patients (*P* for overall = 0.002; *P* for nonlinear = 0.810). The risk of CVD increased when the DII exceeded the median value (Median = 1.934), with significant positive correlation. Additionally, the area under the curve (AUC) for CVD was significantly higher in Model 3 (AUC = 0.750, 95% CI: 0.722–0.775) compared to Model 1 (Delong test: Z = 10.04, *p* < 0.001; Bootstrap Delong test: Z = 9.22, *p* < 0.001) and Model 2 (Delong test: Z = 6.28, *p* < 0.001; Bootstrap Delong test: Z = 6.00, *p* < 0.001). Subtypes of CVD (CHF, CHD, Angina, MI, and Stroke) multi-model analysis presents in Supplementary Figures 6–10.

### Segmented subgroup analysis

Segmented subgroup analysis of 1,437 gout patients were divided into 2 groups, based on the DII (Median = 1.934), revealed a higher risk of CVD in those with DII > 1.934 (OR = 1.33, 95% CI: 1.06–1.65, *p* = 0.012). Among gout patients aged ≤ 60 years with high DII, the risk of CVD was significantly elevated (OR = 2.19, 95% CI: 1.36–3.54, *p* = 0.001), with significant interaction effects (*P* for interaction = 0.012). The prevalence of CVD was slightly higher in females with high DII (38.06% vs. 36.26%) compared to males (OR = 1.46, 95% CI: 0.95–2.25), but this difference was not statistically significant (*p* = 0.087). Gout patients with hypertension and CKD also showed an increased risk of CVD with high DII, although no significant interaction was found (see Table 3).

## Discussion

The association between DII and the occurrence of CVD in patients with gout was explored. Key adjustment variables were screened using machine learning algorithms, revealing a significant positive association between DII and the prevalence of CVD in patients with gout, characterized by a significant linear relationship. Evaluation of the model performance indicated that the fully adjusted model exhibited higher accuracy and discriminative ability. Differences in the risk of CVD in gout patients were observed based on gender and age, but the results remained stability.

DII is an important indicator of dietary regulation of body inflammation, reflects the impact of food components on inflammatory cells, the regulation of oxidative stress, and inflammation mediated by intestinal flora (28, 29). Following the absorption of food components such as cholesterol and fatty acids through the digestive tract, macrophages are activated to release TNF-*α* and IL-6. Concurrently, macrophages phagocytose saturated fat particles, activating intracellular inflammatory signaling pathways, including the NF-κB pathway, which initiates gene transcription, leading to the massive expression of pro-inflammatory cytokines and triggering inflammatory responses (30, 32). Diets containing saturated fats, high levels of alcohol, and caffeine alter the composition and function of the inherently colonized intestinal flora, increasing Enterobacteriaceae and anaerobic bacteria. This results in decreased integrity of the intestinal epithelium, increased intestinal permeability, and enhanced translocation of bacterial endotoxins into the bloodstream, thereby activating the immune response and producing chronic, low-grade inflammation (33). The rational regulation of pro-inflammatory foods and dietary structure interventions warrants further in-depth research and exploration.

The male-to-female prevalence ratio of gout was 2.2:1, and the overall prevalence of CVD was 32.92%, with male-to-female prevalence ratio of approximately 2.3:1. A study conducted at the University of Glasgow, United Kingdom, enrolled 152,663 patients with gout, of whom 120,324 (78.8%) were male. This study found that the risk of CVD was increased by 58% among gout patients compared to the healthcare group (HR 1.88, 95% CI: 1.75–2.02), and 58% increase in the risk of CVD was observed in female with gout (HR = 1.88, 95% CI: 1.75–2.02), despite the higher proportion of male in the cohort (34). A study from the University of Nottingham, UK, including 4,398 patients (66.9% male), found that the incidence of CVD was significantly higher within 30 days of the first diagnosis of gout (HR = 1.55, 95% CI: 1.33–1.83). However, female had a higher incidence of CVD after the diagnosis of gout compared to male, although the statistical difference was not significant (35). These studies and our data were consistent.

DII and the risk of CVD development were positively and linearly correlated. A review of the literature indicates that DII is positively associated with the development of diabetes, hypertension, and hyperuricemia, which are potential risk factors for CVD (17, 36, 37). Covariates screened by machine learning, including age, hypertension, diabetes, BMI, eGFR, BUN, Scr, HbA1c, and UPRO, were identified as correlates of gout comorbidities and their occurrence and progression (38, 39). A study from Peking University in China, involving 7,880 participants from the 2009 China Health and Nutrition Survey (CHNS), found that higher DII was associated with increased risk of hyperuricemia, with 31% reduction in risk in the lowest DII group compared to the highest (40). A study from Zhejiang University of Traditional Chinese Medicine, involving 5,006 participants with CVD, found that as the DII increased, the risk of diabetes and hypertension also increased significantly. Cox proportional hazards modeling revealed that participants in higher DII quartiles exhibited higher CVD mortality (HR = 1.34, 95% CI: 1.21–1.61), showing a positive and linear correlation (41). A Jilin University study, involving 3,930 participants with hyperuricemia, found a positive correlation between the highest quartile of DII levels and the incidence of hyperuricemia (OR = 1.34, 95% CI: 1.13–1.57). With median follow-up of 136 months, 892 deaths were documented, of which 254 were attributed to CVD. Kaplan–Meier curves indicated a 50% higher CVD mortality in participants with higher DII levels (HR = 1.50, 95% CI: 1.00–2.26) (42). A KoGES cohort study of 162,773 healthy participants, with average follow-up of 7.4 years between 2004 and 2013, found that 1,111 participants developed CVD, including 578 males (52.03%) and 533 females (47.97%). Higher DII was associated with increased mortality in males (HR = 1.43, 95% CI: 1.04–1.96) and females (HR = 1.19, 95% CI: 0.85–1.67), with increased risk of developing CVD (43). Although these studies did not directly establish a positive association between DII and the occurrence of CVD in gout patients, they all confirmed the positive association between DII and gout comorbidities and potential risk factors for CVD, offering valuable insights.

We found that the risk of CVD was higher in young and middle-aged gout patients with elevated DII compared to older patients in our subgroup analysis. Gout typically manifests at a younger age in the affected population. Studies have found that the prevalence of gout in males is 2.9 times higher than in females across all age groups, peaking at 7.3 times between the ages of 30 to 34 years (44). Some studies have shown that individuals with gout under the age of 45 have the highest risk of developing CVD after follow-up (HR = 2.22, 95% CI: 1.92–2.57), with excess risk observed across all 12 CVD subtypes investigated (35). Patients diagnosed with gout at or before the age of 40 typically exhibit CVD risk factors, a higher proportion of gout-related family history, lower success in achieving target UA levels, a poorer response to uric acid-lowering medications, and an increased risk of recurrent gout and CVD compared to older patients managed in routine clinical practice (45, 46). Aging and dietary status contribute to differences in dietary inflammatory indices, with reduced physical activity in older adults leading to physiological anorexia and lower intake of food groups (47). A study from the Federal University of Maranhão, Brazil, involving 34,003 healthy participants, found that the DII index was significantly higher in adolescents and adults compared to older adults (1.42 vs. 0.61, *p* < 0.001) (48).

Our study was based on the analysis of U.S. national data with a large, representative sample size, the screening of covariates through machine learning algorithms, and multiple modeling and subgroup analyses. We adhered strictly to the STROBE statement, ensuring the stabilition and reliability of the results. However, the cross-sectional nature of the study presented limitations, including the lack of regular laboratory examinations reviews for the participants and the absence of follow-up on disease progression, as well as the absence of stratified analysis of different types of drug interventions introduced to the participant population, which precluded establishing causality. DII as a complex dietary inflammation-weighting algorithm, may be influenced by confounding factors such as food and water quality. Future clinical patient-based longitudinal studies and randomized controlled trials will further clarify the association between the DII and the development of CVD in gout patients, providing stronger evidence for its use in predicting the risk of CVD in this population.

## Conclusion

Our study suggests a positive, linear association between DII and CVD, as well as its subtypes in gout patients. The predictive performance for CVD and its subtypes was superior in a fully adjusted logistic regression model constructed with machine learning to select variables. Gout patients with high DII and younger age had increased risk of CVD. Future large-scale longitudinal cohort studies are needed to investigate and validate whether a causal relationship exists between DII and CVD, including its subtypes, in gout patients or other populations. The results of this study provide clinicians with both a theoretical and data-driven foundation for the early identification, prevention, and management of CVD in gout patients.

## Data Availability

The datasets presented in this study can be found in online repositories. The names of the repository/repositories and accession number(s) can be found in the article/Supplementary material.
