# Supplementary material for: A cross-sectional exploration of the dietary inflammation index association with cardiovascular disease in gout: application of machine learning algorithms
Source: Front Nutr. 2025 Sep 18;12:1591472. doi: 10.3389/fnut.2025.1591472 (PMC12488447; doi:10.3389/fnut.2025.1591472)
Supplement: Supplementary file 1 [file Supplementary_file_1.DOCX]

Supplementary Material

# Supplementary Tables

**Supplementary Table 1.** **Covariates missingness rates**

| **Variables** | **Freq** | **Miss(n%)** | **Variables** | **Freq** | **Miss** |
| --- | --- | --- | --- | --- | --- |
| Gender | 1,437 | **0 (0.00)** | WBC | 1,367 | **70 (4.87)** |
| Age | 1,437 | **0 (0.00)** | PLT | 1,367 | **70 (4.87)** |
| PIR | 1,315 | **122 (8.49)** | RBC | 1,367 | **70 (4.87)** |
| BMI | 1,408 | **29 (2.02)** | Hb | 1,367 | **70 (4.87)** |
| Drink | 1,320 | **117 (8.14)** | eGFR | 1,335 | **102 (7.10)** |
| Smoke | 1,437 | **0 (0.00)** | UPRO | 1,390 | **47 (3.27)** |
| Hypertension | 1,437 | **0 (0.00)** | BUN | 1,335 | **102 (7.10)** |
| Diabetes | 1,437 | **0 (0.00)** | UA | 1,334 | **103 (7.17)** |
| CHF | 1,437 | **0 (0.00)** | Scr | 1,335 | **102 (7.10)** |
| CHD | 1,437 | **0 (0.00)** | ALB | 1,335 | **102 (7.10)** |
| Angina | 1,437 | **0 (0.00)** | ALT | 1,334 | **103 (7.17)** |
| MI | 1,437 | **0 (0.00)** | AST | 1,331 | **106 (7.38)** |
| Stroke | 1,437 | **0 (0.00)** | GLB | 1,335 | **102 (7.10)** |
| CVD | 1,437 | **0 (0.00)** | GLU | 1,335 | **102 (7.10)** |
| CKD | 1,303 | **134 (9.32)** | HbA1c | 1,364 | **73 (5.08)** |
| Asthma | 1,436 | **1 (0.07)** | Na | 1,334 | **103 (7.17)** |
| COPD | 1,434 | **3 (0.21)** | K | 1,334 | **103 (7.17)** |
| Cancer | 1,436 | **1 (0.07)** | TC | 1,341 | **96 (6.68)** |
| Stone | 1,434 | **3 (0.21)** | TG | 657 | **780 (54.28)** |
| Sleep disorder | 1,437 | **0 (0.00)** | LDL | 636 | **801 (55.74)** |
| SBP Avg | 1,405 | **32 (2.23)** | HDL | 1,341 | **96 (6.68)** |
| DBP Avg | 1,405 | **32 (2.23)** | VitD | 1,322 | **115 (8.00)** |

**Supplementary Table 2.** **Baseline characteristics from**

| **Variable** | **Total** | **Quartiles of DII** | | | | **P** |
| --- | --- | --- | --- | --- | --- | --- |
|  | **(n = 1437)** | **Q1 (n=333)** | **Q2 (n=334)** | **Q3 (n=365)** | **Q4 (n=405)** |  |
| **Age, Mean (SE)** | 60.84 (0.48) | 59.40 (1.08) | 61.21 (0.94) | 60.92 (0.80) | 61.81 (0.88) | 0.142 |
| **Gender, n(%)** |  |  |  |  |  | **<0.001** |
| Male | 1002 (68.77) | 276 (81.19) | 253 (74.84) | 242 (64.48) | 231 (54.69) |  |
| Female | 435 (31.23) | 57 (18.81) | 81 (25.16) | 123 (35.52) | 174 (45.31) |  |
| **BMI, Mean (SE)** | 32.10 (0.33) | 31.23 (0.46) | 32.13 (0.74) | 32.78 (0.49) | 32.25 (0.43) | 0.055 |
| **SBP Avg, Mean (SE)** | 130.63 (0.72) | 129.07 (1.42) | 129.48 (1.34) | 131.02 (1.45) | 132.92 (1.28) | **0.028** |
| **DBP Avg, Mean (SE)** | 71.46 (0.60) | 71.23 (1.00) | 70.75 (1.27) | 72.56 (1.03) | 71.31 (0.83) | 0.600 |
| **Drink, n(%)** | 823 (59.60) | 209 (65.69) | 203 (62.49) | 220 (67.86) | 191 (42.56) | **<0.001** |
| **Smoke, n(%)** | 832 (56.56) | 182 (54.13) | 204 (59.35) | 212 (56.23) | 234 (56.51) | 0.832 |
| **Sleep disorder** | 553 (42.63) | 115 (41.94) | 127 (37.99) | 136 (43.16) | 175 (47.39) | 0.379 |
| **Complications, n(%)** |  |  |  |  |  |  |
| Hypertension | 1051 (68.58) | 232 (64.90) | 234 (65.19) | 276 (73.83) | 309 (70.42) | 0.274 |
| Diabetes | 604 (34.22) | 116 (29.86) | 144 (34.31) | 152 (32.73) | 192 (39.91) | 0.161 |
| CVD | 473 (26.84) | 73 (18.69) | 117 (27.68) | 128 (26.35) | 155 (34.56) | **<0.001** |
| CHF | 191 (9.54) | 25 (4.87) | 45 (7.81) | 50 (10.32) | 71 (15.10) | **<0.001** |
| CHD | 192 (12.64) | 33 (10.10) | 55 (13.55) | 47 (11.35) | 57 (15.54) | 0.328 |
| Angina | 118 (7.75) | 22 (4.68) | 31 (8.82) | 28 (6.86) | 37 (10.61) | 0.099 |
| MI | 204 (11.45) | 30 (6.98) | 52 (13.46) | 53 (11.32) | 69 (13.99) | 0.060 |
| Stroke | 148 (7.44) | 12 (3.15) | 38 (8.83) | 37 (6.12) | 61 (11.59) | **0.005** |
| Asthma | 259 (17.15) | 55 (15.38) | 56 (14.78) | 65 (15.15) | 83 (23.22) | 0.111 |
| COPD | 77 (3.83) | 16 (3.01) | 13 (3.92) | 23 (4.30) | 25 (4.11) | 0.877 |
| CKD | 615 (33.93) | 116 (27.09) | 129 (32.93) | 165 (32.79) | 205 (42.81) | **0.007** |
| Stone | 253 (19.74) | 47 (12.44) | 58 (19.28) | 72 (23.29) | 76 (23.93) | **0.038** |
| Cancer | 290 (22.20) | 64 (15.08) | 72 (21.27) | 64 (26.90) | 90 (25.54) | **0.032** |
| **Laboratory, Mean (SE)** |  |  |  |  |  |  |
| WBC | 7.52 (0.09) | 7.02 (0.15) | 7.58 (0.15) | 7.59 (0.16) | 7.90 (0.16) | **<0.001** |
| PLT, | 229.86 (2.53) | 221.38 (4.09) | 225.27 (5.20) | 226.64 (4.21) | 245.99 (6.62) | **0.003** |
| RBC, | 4.68 (0.02) | 4.77 (0.05) | 4.75 (0.04) | 4.66 (0.04) | 4.53 (0.03) | **<0.001** |
| Hb, | 14.32 (0.08) | 14.68 (0.14) | 14.55 (0.12) | 14.35 (0.11) | 13.72 (0.14) | **<0.001** |
| eGFR, | 75.62 (0.70) | 79.58 (1.34) | 74.03 (1.42) | 75.42 (1.43) | 73.50 (1.49) | **0.014** |
| UPRO, | 98.69 (12.14) | 52.02 (9.47) | 100.58 (22.94) | 114.92 (26.58) | 127.00 (30.14) | **0.020** |
| BUN, | 17.36 (0.25) | 17.05 (0.45) | 17.37 (0.46) | 17.98 (0.52) | 17.03 (0.63) | 0.839 |
| UA, | 6.55 (0.07) | 6.43 (0.12) | 6.44 (0.11) | 6.87 (0.11) | 6.45 (0.14) | 0.375 |
| Scr, | 1.13 (0.03) | 1.07 (0.04) | 1.12 (0.04) | 1.16 (0.05) | 1.17 (0.08) | 0.245 |
| ALB, | 4.19 (0.01) | 4.31 (0.02) | 4.19 (0.03) | 4.16 (0.03) | 4.09 (0.03) | **<0.001** |
| ALT, | 27.05 (0.60) | 28.86 (1.70) | 27.10 (1.01) | 28.46 (1.28) | 23.81 (0.94) | **0.028** |
| AST, | 27.19 (0.41) | 27.84 (1.00) | 26.94 (0.68) | 28.99 (1.12) | 25.03 (0.63) | 0.109 |
| GLB, | 2.88 (0.01) | 2.82 (0.03) | 2.80 (0.03) | 2.95 (0.03) | 2.97 (0.03) | **<0.001** |
| GLU, | 113.99 (1.89) | 111.30 (2.87) | 114.58 (4.18) | 113.36 (3.40) | 116.68 (3.07) | 0.287 |
| HbA1c, | 6.03 (0.05) | 5.95 (0.08) | 6.07 (0.12) | 5.99 (0.07) | 6.13 (0.07) | 0.216 |
| Na, | 139.31 (0.16) | 139.30 (0.19) | 139.37 (0.26) | 139.35 (0.26) | 139.23 (0.21) | 0.790 |
| K, | 4.09 (0.01) | 4.11 (0.03) | 4.07 (0.03) | 4.12 (0.03) | 4.05 (0.02) | 0.177 |
| TC, | 187.69 (1.82) | 186.08 (3.55) | 184.06 (2.52) | 191.16 (4.27) | 189.49 (3.33) | 0.290 |
| TG, | 165.00 (4.32) | 164.90 (6.16) | 154.55 (7.22) | 171.24 (9.64) | 169.35 (6.08) | 0.254 |
| LDL, | 108.25 (1.14) | 108.33 (2.38) | 104.69 (1.68) | 110.26 (2.60) | 109.75 (2.09) | 0.380 |
| HDL, | 47.95 (0.76) | 46.78 (1.00) | 49.36 (1.56) | 48.53 (1.64) | 47.12 (1.24) | 0.972 |

Note: DII: dietary inflammatory index, CVD: cardiovascular disease, SBP Avg: average systolic blood pressure, DBP Avg: average diastolic blood pressure, BMI: body mass index, CHF: congestive heart failure, CHD: coronary heart disease, MI: myocardial infarction, WBC: white blood cell, PLT: platelet, RBC: red blood cell, Hb: hemoglobin, eGFR: estimated glomerular filtration rate, UPRO: urinary protein, BUN: blood urea nitrogen, UA: uric acid, Scr: serum creatinine, ALT: alanine aminotransferase, AST: aspartate aminotransferase, ALB: albumin, GLB: globulin, GLU: glucose, HbA1c: glycated hemoglobin, TC: total cholesterol, TG: triglyceride, LDL: low density lipoprotein, HDL: high density lipoprotein.

P value less than 0.05 indicates a significant difference.

**Supplementary Table 3. Pearson correlation coefficient for correlation matrix**

| **variables** | **env** | **Pearson correlation** | **P value** | **Significance** |
| --- | --- | --- | --- | --- |
| Protein | Energy | 0.714972202660853 | 2.69089835144514e-225 | *** |
| Carbohydrates | Energy | 0.760757400822445 | 1.11689497007525e-271 | *** |
| Carbohydrates | Protein | 0.446597822032272 | 2.25781581032358e-71 | *** |
| Fibre | Energy | -0.473927329 | 2.39105757796412e-81 | *** |
| Fibre | Protein | -0.430178142 | 8.38879419258368e-66 | *** |
| Fibre | Carbohydrates | -0.517429221 | 3.22927381851183e-99 | *** |
| Total fat | Energy | 0.817880927781275 | 0 | *** |
| Total fat | Protein | 0.640132325506543 | 1.67811849105244e-166 | *** |
| Total fat | Carbohydrates | 0.489477609751867 | 1.92861852916542e-87 | *** |
| Total fat | Fibre | -0.372417366 | 1.68184847688733e-48 | *** |
| Saturated fat | Energy | 0.749091514746938 | 6.59056292299993e-259 | *** |
| Saturated fat | Protein | 0.584573593151919 | 1.83101712547741e-132 | *** |
| Saturated fat | Carbohydrates | 0.473757493060756 | 2.77599881189743e-81 | *** |
| Saturated fat | Fibre | -0.292312975 | 1.0515416594779e-29 | *** |
| Saturated fat | Total fat | 0.85492863617874 | 0 | *** |
| MUFA | Energy | -0.786359274 | 1.83465389462808e-302 | *** |
| MUFA | Protein | -0.616570623 | 3.11825693037134e-151 | *** |
| MUFA | Carbohydrates | -0.463004582 | 2.98142315660124e-77 | *** |
| MUFA | Fibre | 0.367406165453358 | 3.71545481783168e-47 | *** |
| MUFA | Total fat | -0.950591832 | 0 | *** |
| MUFA | Saturated fat | -0.786478533 | 1.28913100872526e-302 | *** |
| PUFA | Energy | -0.646610972 | 6.13752951105805e-171 | *** |
| PUFA | Protein | -0.522531751 | 1.73206187875485e-101 | *** |
| PUFA | Carbohydrates | -0.394368714 | 1.12542422032632e-54 | *** |
| PUFA | Fibre | 0.352490594848502 | 2.70443391446765e-43 | *** |
| PUFA | Total fat | -0.805730196 | 0 | *** |
| PUFA | Saturated fat | -0.543772206 | 2.28381782472862e-111 | *** |
| PUFA | MUFA | 0.730515144515241 | 5.73857564267262e-240 | *** |
| Cholesterol | Energy | 0.396646245544105 | 2.41746529171106e-55 | *** |
| Cholesterol | Protein | 0.544770349834142 | 7.52993289346127e-112 | *** |
| Cholesterol | Carbohydrates | 0.145149790595117 | 3.26102950665738e-08 | *** |
| Cholesterol | Fibre | -0.088229609 | 0.000813354808486813 | *** |
| Cholesterol | Total fat | 0.505090511141585 | 6.9601250862774e-94 | *** |
| Cholesterol | Saturated fat | 0.439372662294664 | 6.95741119955652e-69 | *** |
| Cholesterol | MUFA | -0.51031105 | 4.0992964046871e-96 | *** |
| Cholesterol | PUFA | -0.412846093 | 3.01146460209647e-60 | *** |
| VitA | Energy | -0.318623763 | 2.88025097268487e-35 | *** |
| VitA | Protein | -0.328960584 | 1.30461616226028e-37 | *** |
| VitA | Carbohydrates | -0.275475604 | 1.9389160304561e-26 | *** |
| VitA | Fibre | 0.3403618281296 | 2.64812958491462e-40 | *** |
| VitA | Total fat | -0.31636748 | 9.09848038456814e-35 | *** |
| VitA | Saturated fat | -0.338370688 | 7.96878004701177e-40 | *** |
| VitA | MUFA | 0.269991495695441 | 2.00997866312486e-25 | *** |
| VitA | PUFA | 0.239112626567479 | 3.92956603723867e-20 | *** |
| VitA | Cholesterol | -0.215647002 | 1.39675157009775e-16 | *** |
| B carotene | Energy | -0.086778929 | 0.000991429410836188 | *** |
| B carotene | Protein | -0.144941414 | 3.41337211109407e-08 | *** |
| B carotene | Carbohydrates | -0.076812647 | 0.00357323361470522 | ** |
| B carotene | Fibre | 0.274043042506022 | 3.59018409592425e-26 | *** |
| B carotene | Total fat | -0.073090416 | 0.00557142934054449 | ** |
| B carotene | MUFA | 0.064107470053605 | 0.0150756062329808 | * |
| B carotene | PUFA | 0.0865821219193988 | 0.00101819142081088 | ** |
| B carotene | VitA | 0.636763091501134 | 3.0913683379065e-164 | *** |
| Thiamin | Energy | -0.631996137 | 4.43940287126904e-161 | *** |
| Thiamin | Protein | -0.582857774 | 1.62453765667594e-131 | *** |
| Thiamin | Carbohydrates | -0.574411757 | 6.25251496896084e-127 | *** |
| Thiamin | Fibre | 0.491538776883666 | 2.84265657179934e-88 | *** |
| Thiamin | Total fat | -0.522060758 | 2.81690288161247e-101 | *** |
| Thiamin | Saturated fat | -0.501990446 | 1.407915403851e-92 | *** |
| Thiamin | MUFA | 0.487666649570285 | 1.02600545847658e-86 | *** |
| Thiamin | PUFA | 0.444762136667981 | 9.81096030187419e-71 | *** |
| Thiamin | Cholesterol | -0.2153124 | 1.55917940351507e-16 | *** |
| Thiamin | VitA | 0.402604396845212 | 4.08333687820493e-57 | *** |
| Thiamin | B carotene | 0.127768318126321 | 1.17904932227973e-06 | *** |
| Riboflavin | Energy | -0.638466256 | 2.23131914446334e-165 | *** |
| Riboflavin | Protein | -0.617805104 | 5.32171465444708e-152 | *** |
| Riboflavin | Carbohydrates | -0.481847252 | 2.06290199443051e-84 | *** |
| Riboflavin | Fibre | 0.414501953114033 | 9.1627554165018e-61 | *** |
| Riboflavin | Total fat | -0.576580699 | 4.27851617922144e-128 | *** |
| Riboflavin | Saturated fat | -0.562126314 | 1.70642303668076e-120 | *** |
| Riboflavin | MUFA | 0.530000389102043 | 6.99081712810347e-105 | *** |
| Riboflavin | PUFA | 0.457246961525458 | 3.75246746916307e-75 | *** |
| Riboflavin | Cholesterol | -0.371646 | 2.71802486544306e-48 | *** |
| Riboflavin | VitA | 0.522627744919623 | 1.5684681651299e-101 | *** |
| Riboflavin | B carotene | 0.163567840567011 | 4.45617522059527e-10 | *** |
| Riboflavin | Thiamin | 0.706118307116858 | 2.26261216509804e-217 | *** |
| Niacin | Energy | -0.67357153 | 1.26582288589078e-190 | *** |
| Niacin | Protein | -0.753656739 | 8.06714367992133e-264 | *** |
| Niacin | Carbohydrates | -0.504345907 | 1.43722392056594e-93 | *** |
| Niacin | Fibre | 0.44097733072205 | 1.97193143588433e-69 | *** |
| Niacin | Total fat | -0.551033484 | 6.54294198541436e-115 | *** |
| Niacin | Saturated fat | -0.472876808 | 6.01181719861963e-81 | *** |
| Niacin | MUFA | 0.527226173867478 | 1.30362319738925e-103 | *** |
| Niacin | PUFA | 0.491782256652747 | 2.26528524156766e-88 | *** |
| Niacin | Cholesterol | -0.341316448 | 1.55707036954278e-40 | *** |
| Niacin | VitA | 0.338091200931335 | 9.29543516562252e-40 | *** |
| Niacin | B carotene | 0.119878571094027 | 5.19344467126332e-06 | *** |
| Niacin | Thiamin | 0.678125116793144 | 3.70444195724359e-194 | *** |
| Niacin | Riboflavin | 0.665046069815946 | 3.55089169440543e-184 | *** |
| VitB6 | Energy | -0.570898521 | 4.61688159758483e-125 | *** |
| VitB6 | Protein | -0.648407096 | 3.45707790700886e-172 | *** |
| VitB6 | Carbohydrates | -0.435351178 | 1.59156293941924e-67 | *** |
| VitB6 | Fibre | 0.495408251684895 | 7.53774285020645e-90 | *** |
| VitB6 | Total fat | -0.455205779 | 2.03779100048162e-74 | *** |
| VitB6 | Saturated fat | -0.370144975 | 6.89116138726249e-48 | *** |
| VitB6 | MUFA | 0.424713101485686 | 5.13475394338536e-64 | *** |
| VitB6 | PUFA | 0.41367914897602 | 1.65640107487465e-60 | *** |
| VitB6 | Cholesterol | -0.304852552 | 2.76680851848904e-32 | *** |
| VitB6 | VitA | 0.438965698033891 | 9.56858572046324e-69 | *** |
| VitB6 | B carotene | 0.221921416987562 | 1.71582621187484e-17 | *** |
| VitB6 | Thiamin | 0.589738344089457 | 2.36910280304452e-135 | *** |
| VitB6 | Riboflavin | 0.651406640109051 | 2.71270687251963e-174 | *** |
| VitB6 | Niacin | 0.773731361136277 | 9.00857144975425e-287 | *** |
| folic acid | Energy | -0.301544142 | 1.36485250041248e-31 | *** |
| folic acid | Protein | -0.247367084 | 1.77797055923288e-21 | *** |
| folic acid | Carbohydrates | -0.365572421 | 1.13756177254845e-46 | *** |
| folic acid | Fibre | 0.298549626354701 | 5.68472474953375e-31 | *** |
| folic acid | Total fat | -0.211408423 | 5.55244051876021e-16 | *** |
| folic acid | Saturated fat | -0.231109851 | 7.07963866828768e-19 | *** |
| folic acid | MUFA | 0.174299483653829 | 2.88073834681716e-11 | *** |
| folic acid | PUFA | 0.179762576572212 | 6.67425677288267e-12 | *** |
| folic acid | VitA | 0.335859551104011 | 3.16089489308655e-39 | *** |
| folic acid | Thiamin | 0.615467681919545 | 1.50337645141344e-150 | *** |
| folic acid | Riboflavin | 0.448764669016909 | 3.94016471015526e-72 | *** |
| folic acid | Niacin | 0.427179946012756 | 8.09178902873376e-65 | *** |
| folic acid | VitB6 | 0.374223623681247 | 5.43766835555746e-49 | *** |
| VitB12 | Energy | 0.410568770481906 | 1.53016637613094e-59 | *** |
| VitB12 | Protein | 0.499067383607981 | 2.33188156855813e-91 | *** |
| VitB12 | Carbohydrates | 0.286238346423585 | 1.68251845155771e-28 | *** |
| VitB12 | Fibre | -0.222534579 | 1.39307638341438e-17 | *** |
| VitB12 | Total fat | 0.386616131120506 | 1.93207957529178e-52 | *** |
| VitB12 | Saturated fat | 0.403075327203352 | 2.9469954947891e-57 | *** |
| VitB12 | MUFA | -0.35351923 | 1.48701127128285e-43 | *** |
| VitB12 | PUFA | -0.264558461 | 1.93401920278359e-24 | *** |
| VitB12 | Cholesterol | 0.275117510635658 | 2.26250503557523e-26 | *** |
| VitB12 | VitA | -0.428724189 | 2.52517242885159e-65 | *** |
| VitB12 | B carotene | -0.072097022 | 0.00625285129505141 | ** |
| VitB12 | Thiamin | -0.453531395 | 8.09530866823364e-74 | *** |
| VitB12 | Riboflavin | -0.580769661 | 2.27443205952369e-130 | *** |
| VitB12 | Niacin | -0.537374189 | 2.56564876708144e-108 | *** |
| VitB12 | VitB6 | -0.57000975 | 1.35961912716123e-124 | *** |
| VitB12 | folic acid | -0.416433221 | 2.2680167268884e-61 | *** |
| VitC | Energy | -0.169675463 | 9.58120098972125e-11 | *** |
| VitC | Protein | -0.143837651 | 4.34258884063447e-08 | *** |
| VitC | Carbohydrates | -0.224813416 | 6.38666439373587e-18 | *** |
| VitC | Fibre | 0.312064221640825 | 7.9402153444469e-34 | *** |
| VitC | Total fat | -0.073550802 | 0.00527894401414714 | ** |
| VitC | MUFA | 0.0708504964236943 | 0.00721347364878097 | ** |
| VitC | PUFA | 0.0770061032320493 | 0.00348990064755783 | ** |
| VitC | Cholesterol | -0.067370069 | 0.0106331221483476 | * |
| VitC | VitA | 0.250309332497003 | 5.73454116063021e-22 | *** |
| VitC | B carotene | 0.258318547047331 | 2.44290215534661e-23 | *** |
| VitC | Thiamin | 0.195727677523926 | 7.11882454583192e-14 | *** |
| VitC | Riboflavin | 0.173015635518209 | 4.03502645838754e-11 | *** |
| VitC | Niacin | 0.155328054177181 | 3.23951630604128e-09 | *** |
| VitC | VitB6 | 0.312351425856364 | 6.87893405737599e-34 | *** |
| VitC | folic acid | 0.0766275251216682 | 0.00365466533799784 | ** |
| VitC | VitB12 | -0.088080626 | 0.000830172540113637 | *** |
| VitD | Energy | -0.261391074 | 7.06738938848885e-24 | *** |
| VitD | Protein | -0.300535276 | 2.2113479601519e-31 | *** |
| VitD | Carbohydrates | -0.186392403 | 1.06313835273497e-12 | *** |
| VitD | Fibre | 0.113340968276349 | 1.65745618455754e-05 | *** |
| VitD | Total fat | -0.237579559 | 6.89463994317871e-20 | *** |
| VitD | Saturated fat | -0.238338716 | 5.22177608012577e-20 | *** |
| VitD | MUFA | 0.201583820506133 | 1.21677207981865e-14 | *** |
| VitD | PUFA | 0.186837811995553 | 9.37390628406557e-13 | *** |
| VitD | Cholesterol | -0.189493072 | 4.39752572550299e-13 | *** |
| VitD | VitA | 0.3816922524634 | 4.72561442081283e-51 | *** |
| VitD | B carotene | 0.0777398400594845 | 0.00318964982697926 | ** |
| VitD | Thiamin | 0.336490509420661 | 2.23857000421299e-39 | *** |
| VitD | Riboflavin | 0.44058854096819 | 2.67810374636785e-69 | *** |
| VitD | Niacin | 0.278050566628621 | 6.34761968926815e-27 | *** |
| VitD | VitB6 | 0.33926937405387 | 4.85154195979643e-40 | *** |
| VitD | folic acid | 0.263921056522566 | 2.51381771826236e-24 | *** |
| VitD | VitB12 | -0.571580906 | 2.01016112097436e-125 | *** |
| VitD | VitC | 0.116285371362444 | 9.90282032865533e-06 | *** |
| VitE | Energy | -0.574437976 | 6.05378272771159e-127 | *** |
| VitE | Protein | -0.480413294 | 7.50316590379239e-84 | *** |
| VitE | Carbohydrates | -0.394931643 | 7.7038386963659e-55 | *** |
| VitE | Fibre | 0.500137725910735 | 8.36891520214779e-92 | *** |
| VitE | Total fat | -0.614692341 | 4.52572592074256e-150 | *** |
| VitE | Saturated fat | -0.472644969 | 7.36521506311101e-81 | *** |
| VitE | MUFA | 0.598954616123104 | 1.22824873427338e-140 | *** |
| VitE | PUFA | 0.60449248183282 | 6.75838108973134e-144 | *** |
| VitE | Cholesterol | -0.285449082 | 2.40021538761591e-28 | *** |
| VitE | VitA | 0.390283085210986 | 1.72377821061338e-53 | *** |
| VitE | B carotene | 0.218243887270374 | 5.91038238436004e-17 | *** |
| VitE | Thiamin | 0.477035293613741 | 1.5337417964662e-82 | *** |
| VitE | Riboflavin | 0.50393264181577 | 2.14766320308749e-93 | *** |
| VitE | Niacin | 0.510829880650646 | 2.44890349012641e-96 | *** |
| VitE | VitB6 | 0.484989615898046 | 1.19192837514309e-85 | *** |
| VitE | folic acid | 0.294212406132833 | 4.35755081372733e-30 | *** |
| VitE | VitB12 | -0.350010514 | 1.13348988071632e-42 | *** |
| VitE | VitC | 0.228247396664623 | 1.94015100274491e-18 | *** |
| VitE | VitD | 0.242831404861579 | 9.88301856184312e-21 | *** |
| Mg | Energy | -0.679791426 | 1.81793798367825e-195 | *** |
| Mg | Protein | -0.630145413 | 7.21427492725921e-160 | *** |
| Mg | Carbohydrates | -0.558367678 | 1.40504562217193e-118 | *** |
| Mg | Fibre | 0.740268957269213 | 1.03972084820023e-249 | *** |
| Mg | Total fat | -0.533043437 | 2.73602367669527e-106 | *** |
| Mg | Saturated fat | -0.443246548 | 3.27746630007834e-70 | *** |
| Mg | MUFA | 0.509522221819932 | 8.9565346332011e-96 | *** |
| Mg | PUFA | 0.48100610678145 | 4.40292886294045e-84 | *** |
| Mg | Cholesterol | -0.217336536 | 7.99212225851769e-17 | *** |
| Mg | VitA | 0.438390243062705 | 1.50046654996595e-68 | *** |
| Mg | B carotene | 0.260098698755083 | 1.19316239679538e-23 | *** |
| Mg | Thiamin | 0.602082255905986 | 1.8042681643311e-142 | *** |
| Mg | Riboflavin | 0.64298736511971 | 1.91794840196988e-168 | *** |
| Mg | Niacin | 0.658401200780845 | 2.69038824501711e-179 | *** |
| Mg | VitB6 | 0.671441888995145 | 5.41135443310084e-189 | *** |
| Mg | folic acid | 0.314940619209041 | 1.87352496341405e-34 | *** |
| Mg | VitB12 | -0.409903215 | 2.4548633719804e-59 | *** |
| Mg | VitC | 0.306667802893793 | 1.14251464769879e-32 | *** |
| Mg | VitD | 0.329215056955171 | 1.13925838185819e-37 | *** |
| Mg | VitE | 0.604597169157785 | 5.85609268408555e-144 | *** |
| Fe | Energy | 0.614333707456397 | 7.52701808531881e-150 | *** |
| Fe | Protein | 0.571143634635059 | 3.42556540899852e-125 | *** |
| Fe | Carbohydrates | 0.564478101311178 | 1.04936898838149e-121 | *** |
| Fe | Fibre | -0.556563699 | 1.14398134263704e-117 | *** |
| Fe | Total fat | 0.491950338270515 | 1.93646086086812e-88 | *** |
| Fe | Saturated fat | 0.47059939666497 | 4.38814617054664e-80 | *** |
| Fe | MUFA | -0.465985474 | 2.35151301654823e-78 | *** |
| Fe | PUFA | -0.396709881 | 2.31537786677078e-55 | *** |
| Fe | Cholesterol | 0.237818790449555 | 6.31718075763543e-20 | *** |
| Fe | VitA | -0.462348303 | 5.19773650509195e-77 | *** |
| Fe | B carotene | -0.160299344 | 9.9094814234069e-10 | *** |
| Fe | Thiamin | -0.761443993 | 1.87738930231718e-272 | *** |
| Fe | Riboflavin | -0.688212656 | 3.2197572167929e-202 | *** |
| Fe | Niacin | -0.641262522 | 2.87436597303778e-167 | *** |
| Fe | VitB6 | -0.622484912 | 6.0789907353143e-155 | *** |
| Fe | folic acid | -0.611024027 | 7.98295103353438e-148 | *** |
| Fe | VitB12 | 0.537596721872343 | 2.01462483644209e-108 | *** |
| Fe | VitC | -0.175647581 | 2.01673271800968e-11 | *** |
| Fe | VitD | -0.297512293 | 9.28219852463053e-31 | *** |
| Fe | VitE | -0.477841506 | 7.48708478852889e-83 | *** |
| Fe | Mg | -0.641065909 | 3.9091206291237e-167 | *** |
| Zinc | Energy | -0.629560791 | 1.73373683464657e-159 | *** |
| Zinc | Protein | -0.724398243 | 4.49746447371457e-234 | *** |
| Zinc | Carbohydrates | -0.46363825 | 1.741203039257e-77 | *** |
| Zinc | Fibre | 0.476072677975087 | 3.60180885643371e-82 | *** |
| Zinc | Total fat | -0.558747818 | 9.01704636542182e-119 | *** |
| Zinc | Saturated fat | -0.557224409 | 5.31510455661741e-118 | *** |
| Zinc | MUFA | 0.544489080164864 | 1.02977645428625e-111 | *** |
| Zinc | PUFA | 0.381710041152309 | 4.67180786751894e-51 | *** |
| Zinc | Cholesterol | -0.32407747 | 1.71456814992933e-36 | *** |
| Zinc | VitA | 0.383301186997091 | 1.67257989530731e-51 | *** |
| Zinc | B carotene | 0.163617725685538 | 4.40159601101559e-10 | *** |
| Zinc | Thiamin | 0.59279972267581 | 4.34411813999094e-137 | *** |
| Zinc | Riboflavin | 0.607427726466282 | 1.19145373658735e-145 | *** |
| Zinc | Niacin | 0.610246675534846 | 2.36827425372938e-147 | *** |
| Zinc | VitB6 | 0.614886522093263 | 3.43514467880158e-150 | *** |
| Zinc | folic acid | 0.41177344395956 | 6.48598381267496e-60 | *** |
| Zinc | VitB12 | -0.574304338 | 7.13698524532968e-127 | *** |
| Zinc | VitC | 0.173292656917537 | 3.75286397393623e-11 | *** |
| Zinc | VitD | 0.261080108553644 | 8.01863947943345e-24 | *** |
| Zinc | VitE | 0.447922885510667 | 7.77506125972685e-72 | *** |
| Zinc | Mg | 0.636920742139597 | 2.42540434092791e-164 | *** |
| Zinc | Fe | -0.698009055 | 2.27430940926541e-210 | *** |
| Selenium | Energy | -0.556101349 | 1.95404670313026e-117 | *** |
| Selenium | Protein | -0.697627565 | 4.79135234338904e-210 | *** |
| Selenium | Carbohydrates | -0.353987992 | 1.13140295185705e-43 | *** |
| Selenium | Fibre | 0.294881819657708 | 3.18944992137509e-30 | *** |
| Selenium | Total fat | -0.571761991 | 1.61160503263909e-125 | *** |
| Selenium | Saturated fat | -0.466688402 | 1.28716343440289e-78 | *** |
| Selenium | MUFA | 0.527256732658888 | 1.26246686628491e-103 | *** |
| Selenium | PUFA | 0.531577038867991 | 1.30954843778272e-105 | *** |
| Selenium | Cholesterol | -0.538351988 | 8.85589095578181e-109 | *** |
| Selenium | VitA | 0.260672937305749 | 9.4579726980608e-24 | *** |
| Selenium | B carotene | 0.118258084462566 | 6.96415695049468e-06 | *** |
| Selenium | Thiamin | 0.520780001981793 | 1.05299715908384e-100 | *** |
| Selenium | Riboflavin | 0.52638174203817 | 3.15918199403497e-103 | *** |
| Selenium | Niacin | 0.588248007308221 | 1.63427190967924e-134 | *** |
| Selenium | VitB6 | 0.501220592740703 | 2.95672481007139e-92 | *** |
| Selenium | folic acid | 0.216396636729796 | 1.090919207423e-16 | *** |
| Selenium | VitB12 | -0.375389951 | 2.61283288825347e-49 | *** |
| Selenium | VitC | 0.106767309357062 | 5.00514986621743e-05 | *** |
| Selenium | VitD | 0.263910677298816 | 2.52455869622017e-24 | *** |
| Selenium | VitE | 0.375883191560952 | 1.91473579820023e-49 | *** |
| Selenium | Mg | 0.472930250005994 | 5.73679726075115e-81 | *** |
| Selenium | Fe | -0.452128042 | 2.55721649313243e-73 | *** |
| Selenium | Zinc | 0.513341306729653 | 1.99745061344393e-97 | *** |
| Caffeine | Energy | -0.183430371 | 2.43621220649955e-12 | *** |
| Caffeine | Protein | -0.123381162 | 2.71913206235786e-06 | *** |
| Caffeine | Carbohydrates | -0.147699635 | 1.85540093151604e-08 | *** |
| Caffeine | Fibre | 0.0762592545636122 | 0.0038216927509941 | ** |
| Caffeine | Total fat | -0.15740115 | 1.98576904717272e-09 | *** |
| Caffeine | Saturated fat | -0.153670089 | 4.76917757142278e-09 | *** |
| Caffeine | MUFA | 0.156313718163573 | 2.56905158832264e-09 | *** |
| Caffeine | PUFA | 0.1504242680556 | 1.00477935327919e-08 | *** |
| Caffeine | Thiamin | 0.146913156874001 | 2.21018967705774e-08 | *** |
| Caffeine | Riboflavin | 0.354990076451734 | 6.2979972774793e-44 | *** |
| Caffeine | Niacin | 0.168797018139061 | 1.1993813410978e-10 | *** |
| Caffeine | VitC | -0.069428328 | 0.00846939202791929 | ** |
| Caffeine | VitE | 0.127334566197713 | 1.28219168565792e-06 | *** |
| Caffeine | Mg | 0.200643523521266 | 1.62179660538523e-14 | *** |
| Caffeine | Fe | -0.093552244 | 0.000383679776398133 | *** |
| Caffeine | Zinc | 0.0959337823558224 | 0.000270659781912673 | *** |
| Caffeine | Selenium | 0.0875615064293619 | 0.000891323067585434 | *** |
| Alcohol | Energy | -0.228928335 | 1.52834157435126e-18 | *** |
| Alcohol | Protein | -0.11912249 | 5.95810451872421e-06 | *** |
| Alcohol | Total fat | -0.101814522 | 0.000110519488339952 | *** |
| Alcohol | Saturated fat | -0.07280943 | 0.00575704144513977 | ** |
| Alcohol | MUFA | 0.109455138578235 | 3.20920480405662e-05 | *** |
| Alcohol | PUFA | 0.101438905447336 | 0.000117196686395734 | *** |
| Alcohol | Cholesterol | -0.067550945 | 0.0104249965389697 | * |
| Alcohol | Riboflavin | 0.127905202702685 | 1.14818910148756e-06 | *** |
| Alcohol | Niacin | 0.189066598553412 | 4.96968536951074e-13 | *** |
| Alcohol | VitB6 | 0.189941471354551 | 3.86565268754244e-13 | *** |
| Alcohol | VitB12 | -0.068868827 | 0.009014765355796 | ** |
| Alcohol | VitE | 0.0667236001401247 | 0.011407476016952 | * |
| Alcohol | Mg | 0.19973560570006 | 2.13749043931899e-14 | *** |
| Alcohol | Zinc | 0.0830121952015657 | 0.00163546213063343 | ** |
| Alcohol | Selenium | 0.0995416553965179 | 0.000157137355839882 | *** |
| n3 fatty acids | Energy | -0.148579669 | 1.52380961403259e-08 | *** |
| n3 fatty acids | Protein | -0.248596845 | 1.10996666971799e-21 | *** |
| n3 fatty acids | Fibre | 0.053769337769437 | 0.0415522342363222 | * |
| n3 fatty acids | Total fat | -0.134935329 | 2.83430082090204e-07 | *** |
| n3 fatty acids | MUFA | 0.13364610119969 | 3.6831627595626e-07 | *** |
| n3 fatty acids | PUFA | 0.171880280346774 | 5.42429632137064e-11 | *** |
| n3 fatty acids | Cholesterol | -0.18215888 | 3.4631926392498e-12 | *** |
| n3 fatty acids | VitA | 0.133038020671928 | 4.16405420122329e-07 | *** |
| n3 fatty acids | B carotene | 0.0838341922168348 | 0.00146867227364102 | ** |
| n3 fatty acids | Thiamin | 0.11488689248558 | 1.26669829249338e-05 | *** |
| n3 fatty acids | Riboflavin | 0.0859170230675872 | 0.0011136535471758 | ** |
| n3 fatty acids | Niacin | 0.214387698343646 | 2.11115270849755e-16 | *** |
| n3 fatty acids | VitB6 | 0.166839165979543 | 1.97014413579712e-10 | *** |
| n3 fatty acids | VitB12 | -0.389535333 | 2.82868308300974e-53 | *** |
| n3 fatty acids | VitD | 0.44639744077589 | 2.65170201426566e-71 | *** |
| n3 fatty acids | VitE | 0.207290846932495 | 2.06356120508666e-15 | *** |
| n3 fatty acids | Mg | 0.18592085180425 | 1.2142845316142e-12 | *** |
| n3 fatty acids | Fe | -0.080650579 | 0.00221625954306971 | ** |
| n3 fatty acids | Zinc | 0.0837931611987379 | 0.00147661170160103 | ** |
| n3 fatty acids | Selenium | 0.230132668555267 | 1.0003267388971e-18 | *** |
| n3 fatty acids | Alcohol | 0.10093306469373 | 0.000126790866455186 | *** |
| n6 fatty acids | Energy | -0.656057259 | 1.3227310468925e-177 | *** |
| n6 fatty acids | Protein | -0.529643748 | 1.0198499933984e-104 | *** |
| n6 fatty acids | Carbohydrates | -0.408234986 | 7.99001857288759e-59 | *** |
| n6 fatty acids | Fibre | 0.363160864989507 | 4.90077222618223e-46 | *** |
| n6 fatty acids | Total fat | -0.815222646 | 0 | *** |
| n6 fatty acids | Saturated fat | -0.560243996 | 1.56490032404371e-119 | *** |
| n6 fatty acids | MUFA | 0.737974382960495 | 2.22795139887502e-247 | *** |
| n6 fatty acids | PUFA | 0.981845831646706 | 0 | *** |
| n6 fatty acids | Cholesterol | -0.41712148 | 1.37589440166728e-61 | *** |
| n6 fatty acids | VitA | 0.249013283798147 | 9.45725601299358e-22 | *** |
| n6 fatty acids | B carotene | 0.0953978946963713 | 0.000292970465322969 | *** |
| n6 fatty acids | Thiamin | 0.461439308919239 | 1.12018362681584e-76 | *** |
| n6 fatty acids | Riboflavin | 0.474551099809652 | 1.38093310761607e-81 | *** |
| n6 fatty acids | Niacin | 0.504396867486827 | 1.36771990753978e-93 | *** |
| n6 fatty acids | VitB6 | 0.429106451063065 | 1.8909937541504e-65 | *** |
| n6 fatty acids | folic acid | 0.191028804981173 | 2.82416945167278e-13 | *** |
| n6 fatty acids | VitB12 | -0.269898528 | 2.0902963882413e-25 | *** |
| n6 fatty acids | VitC | 0.0761215549156623 | 0.00388590487218382 | ** |
| n6 fatty acids | VitD | 0.182449639335193 | 3.19624764660914e-12 | *** |
| n6 fatty acids | VitE | 0.610980957740753 | 8.4793807366064e-148 | *** |
| n6 fatty acids | Mg | 0.495976404897411 | 4.40612885557307e-90 | *** |
| n6 fatty acids | Fe | -0.413458334 | 1.94111602826307e-60 | *** |
| n6 fatty acids | Zinc | 0.402380786058961 | 4.76637128146057e-57 | *** |
| n6 fatty acids | Selenium | 0.560095740836984 | 1.86219413142737e-119 | *** |
| n6 fatty acids | Caffeine | 0.146606285115854 | 2.36576520751322e-08 | *** |
| n6 fatty acids | Alcohol | 0.0949496457488081 | 0.000312942373184443 | *** |
| n6 fatty acids | n3 fatty acids | 0.153820688455895 | 4.60533163504755e-09 | *** |

**Supplementary table 4. Feature selection of DII with CVD**

| **Machine Learning** | **CVD** | **CHF** | **CHD** | **Angina** | **MI** | **Stroke** |
| --- | --- | --- | --- | --- | --- | --- |
| **Boruta** | TC, Age, eGFR, BUN, Hypertension, PLT, Scr, UPRO, HbA1c, Diabetes | eGFR, Scr, BUN, UPRO, PLT, Age, HbA1c, COPD, GLU, CKD | eGFR, Age, BUN, Scr, TC, Hb, UPRO, HDL, CKD, ALT | eGFR, BUN, Scr, Hb, RBC, Age, UPRO, HbA1c, DBP Avg, CKD | eGFR, TC, BUN, Scr, Age, Hb, RBC, PLT, CKD, SBP Avg | eGFR, Scr, BUN, Age, RBC, UPRO, ALT, Hb, CKD, PLT |
| **Random forest** | TC, eGFR, Age, PLT, UPRO, Scr, BUN, WBC, SBP Avg | K, BMI, SBP Avg, TC, HbA1c, UPRO, Scr, PLT, eGFR, BUN | RBC, GLU, Scr, K, HDL, UPRO, Hb, eGFR, PLT, TC | UPRO, HbA1c, Hb, WBC, SBP Avg, eGFR, BMI, TC, PLT, HDL | DBP Avg, RBC, HDL, Scr, BUN, SBP Avg, eGFR, UPRO, PLT, TC | BUN, Scr, GLU, Hb, RBC, UPRO, SBP Avg, PLT, TC, eGFR |
| **Model 3** | Age, Hypertension, Diabetes, BMI, eGFR, BUN, Scr, HbA1c, PLT, UPRO, WBC, SBP Avg, TC | Age, CKD, eGFR, BUN, Scr, HbA1c, GLU, UPRO, PLT, COPD, K, TC, SBP Avg | Age, eGFR, BUN, Scr, GLU, TC, Hb, UPRO, HDL, CKD, ALT, RBC, K, PLT | Age, CKD, BMI, eGFR, BUN, Scr, HbA1c, Hb, RBC, UPRO, DBP Avg, WBC, TC, PLT, HDL, SBP Avg | Age, CKD, eGFR, BUN, Scr, TC, Hb, RBC, PLT, HDL, UPRO, DBP Avg, SBP Avg | Age, CKD, eGFR, BUN, Scr, GLU, RBC, UPRO, ALT, Hb, PLT, TC, SBP Avg |

Note: CVD: cardiovascular disease, SBP Avg: average systolic blood pressure, DBP Avg: average diastolic blood pressure, BMI: body mass index, CHF: congestive heart failure, CHD: coronary heart disease, MI: myocardial infarction, WBC: white blood cell, PLT: platelet, RBC: red blood cell, Hb: hemoglobin, eGFR: estimated glomerular filtration rate, UPRO: urinary protein, BUN: blood urea nitrogen, UA: uric acid, Scr: serum creatinine, ALT: alanine aminotransferase, AST: aspartate aminotransferase, ALB: albumin, GLB: globulin, GLU: glucose, HbA1c: glycated hemoglobin, TC: total cholesterol, TG: triglyceride, LDL: low density lipoprotein, HDL: high density lipoprotein.

# Supplementary Tables

**
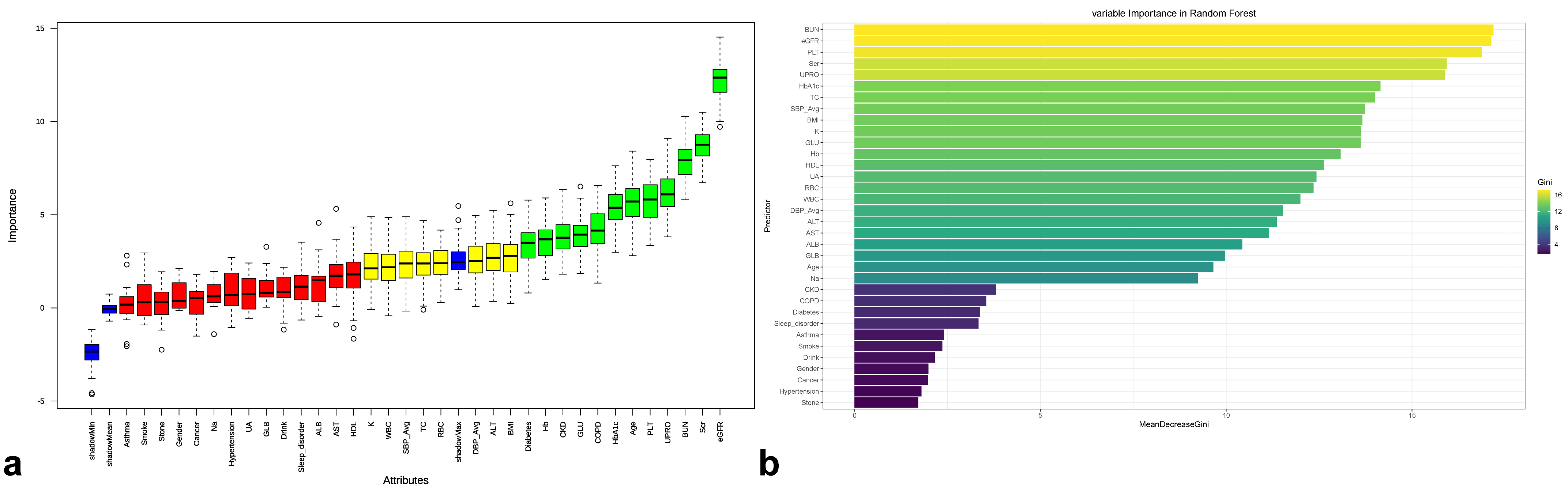
**

**Supplementary Figure 1. Ranking of variable importance based on Boruta(a) and Random Forest(b) algorithms of CHF**

**
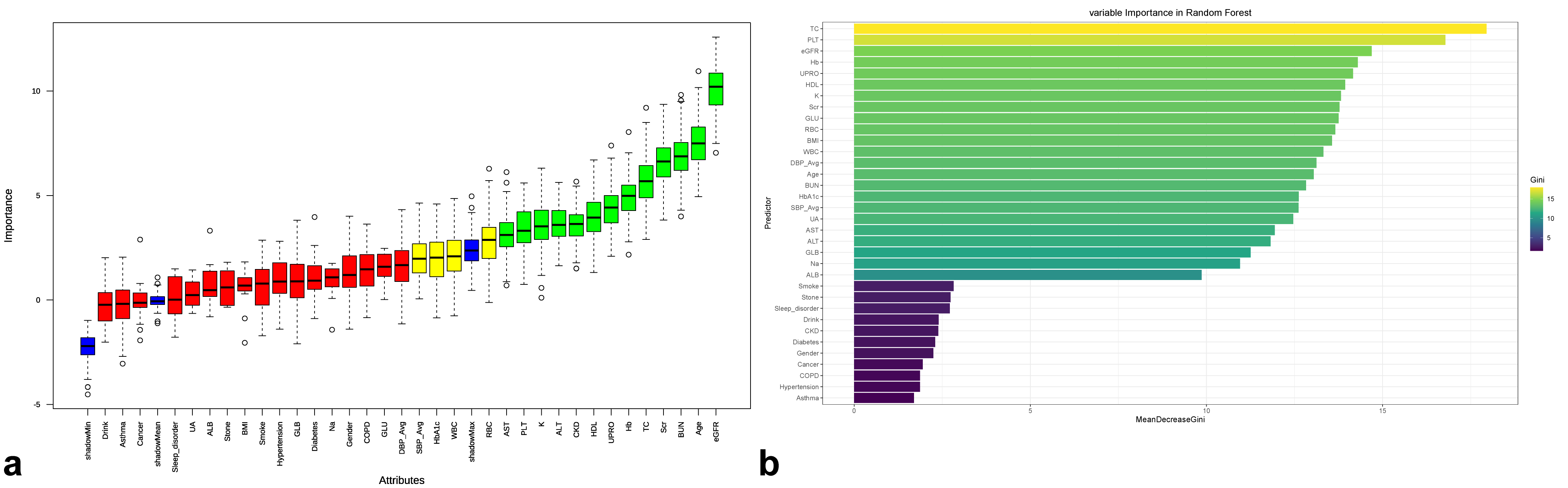
**

**Supplementary Figure 2. Ranking of variable importance based on Boruta(a) and Random Forest(b)**

**algorithms of CHD**

**
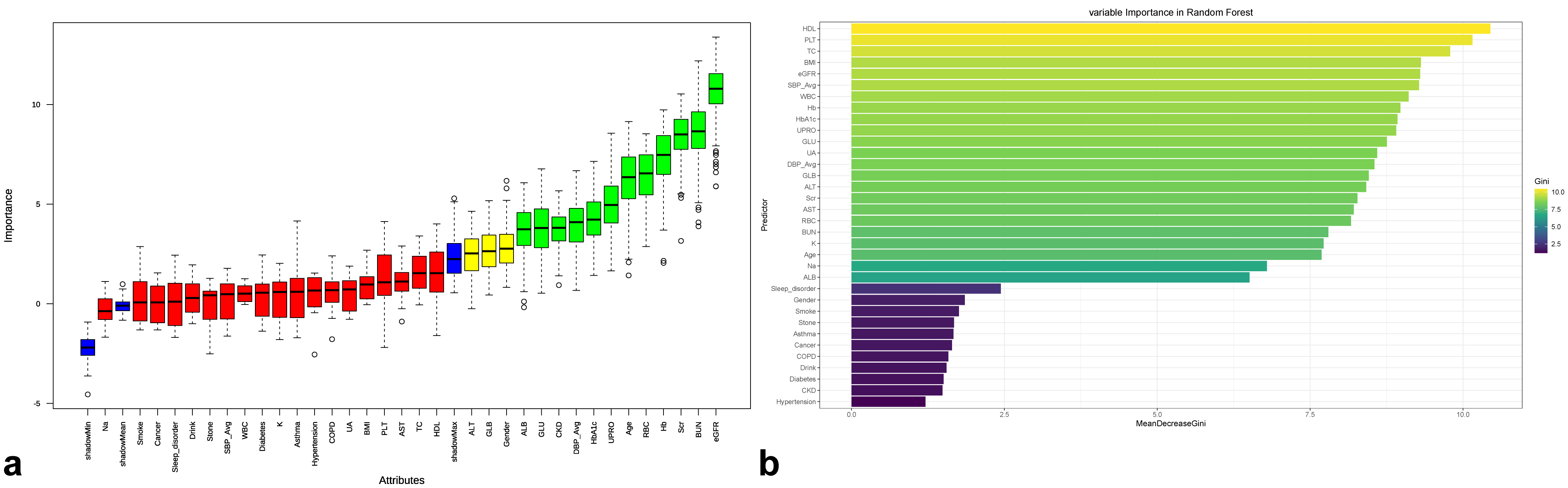
**

**Supplementary Figure 3. Ranking of variable importance based on Boruta(a) and Random Forest(b) algorithms of Angina**

**
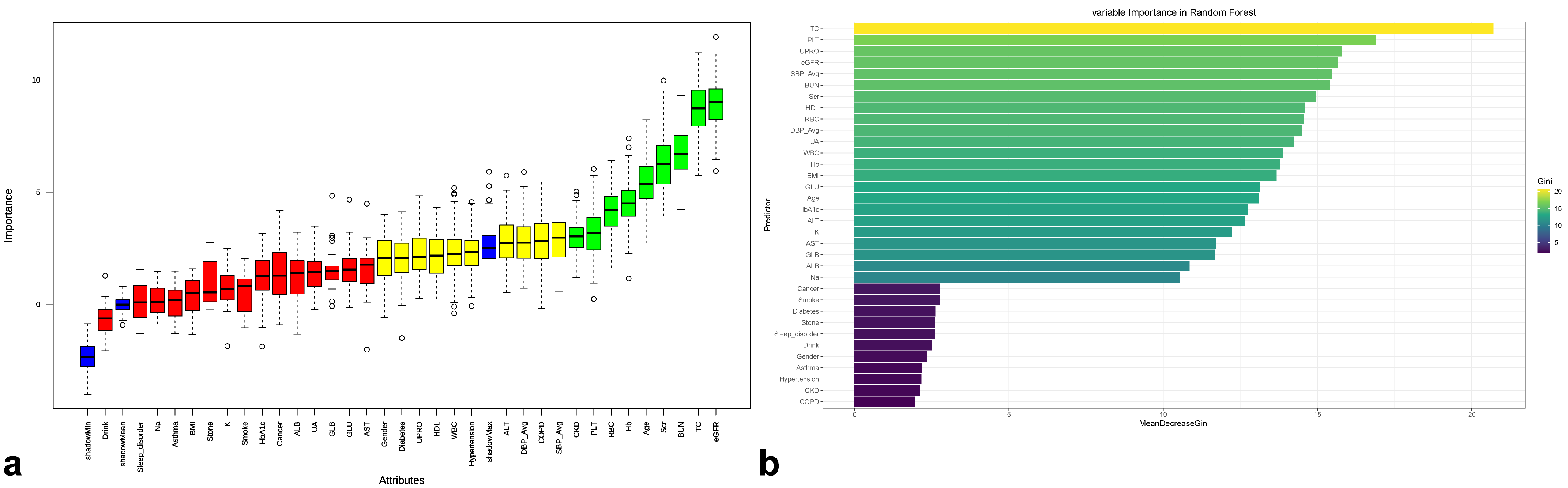
**

**Supplementary Figure 4. Ranking of variable importance based on Boruta(a) and Random Forest(b) algorithms of MI**

**
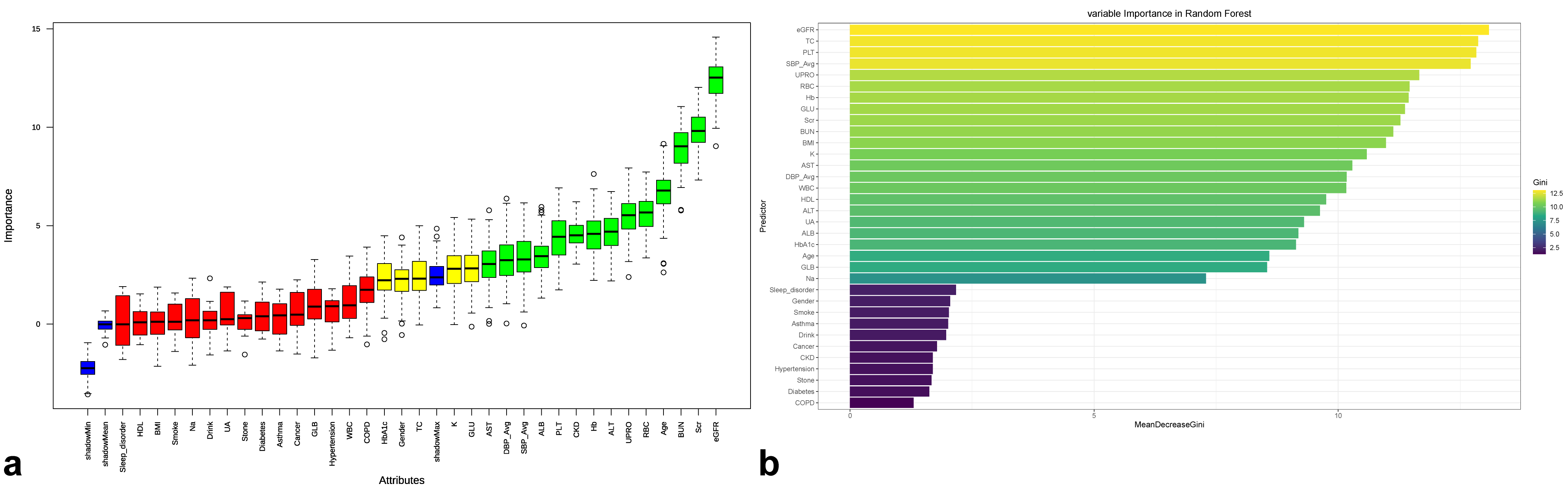
**

**Supplementary Figure 5. Ranking of variable importance based on Boruta(a) and Random Forest(b) algorithms of Stroke**

**
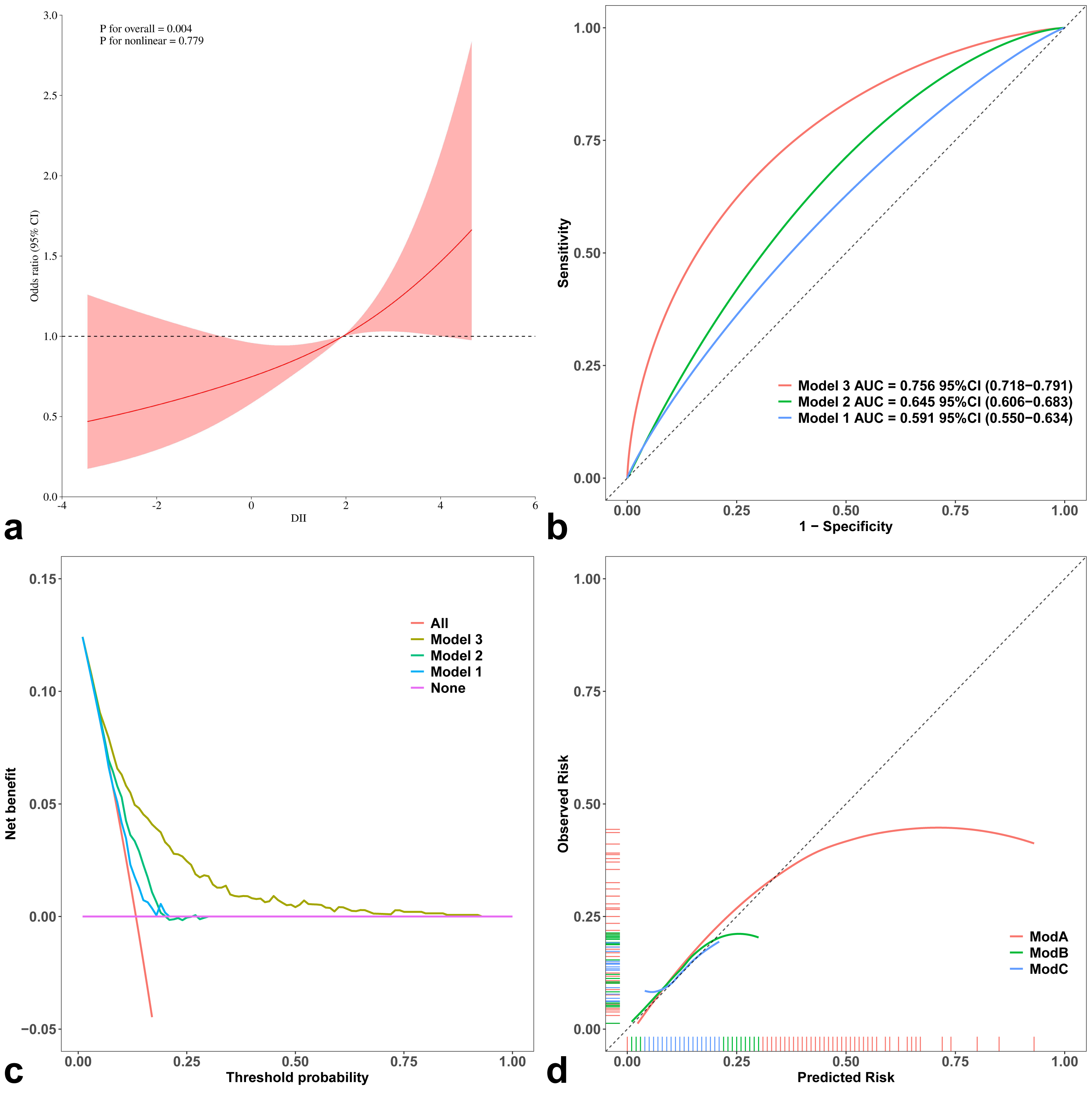
**

**Supplementary Figure 6. Performance evaluation of Multi-model incorporating confounders with RCS(a), ROC(b), DCA(c) and calibration curve(d) of CHF**

**
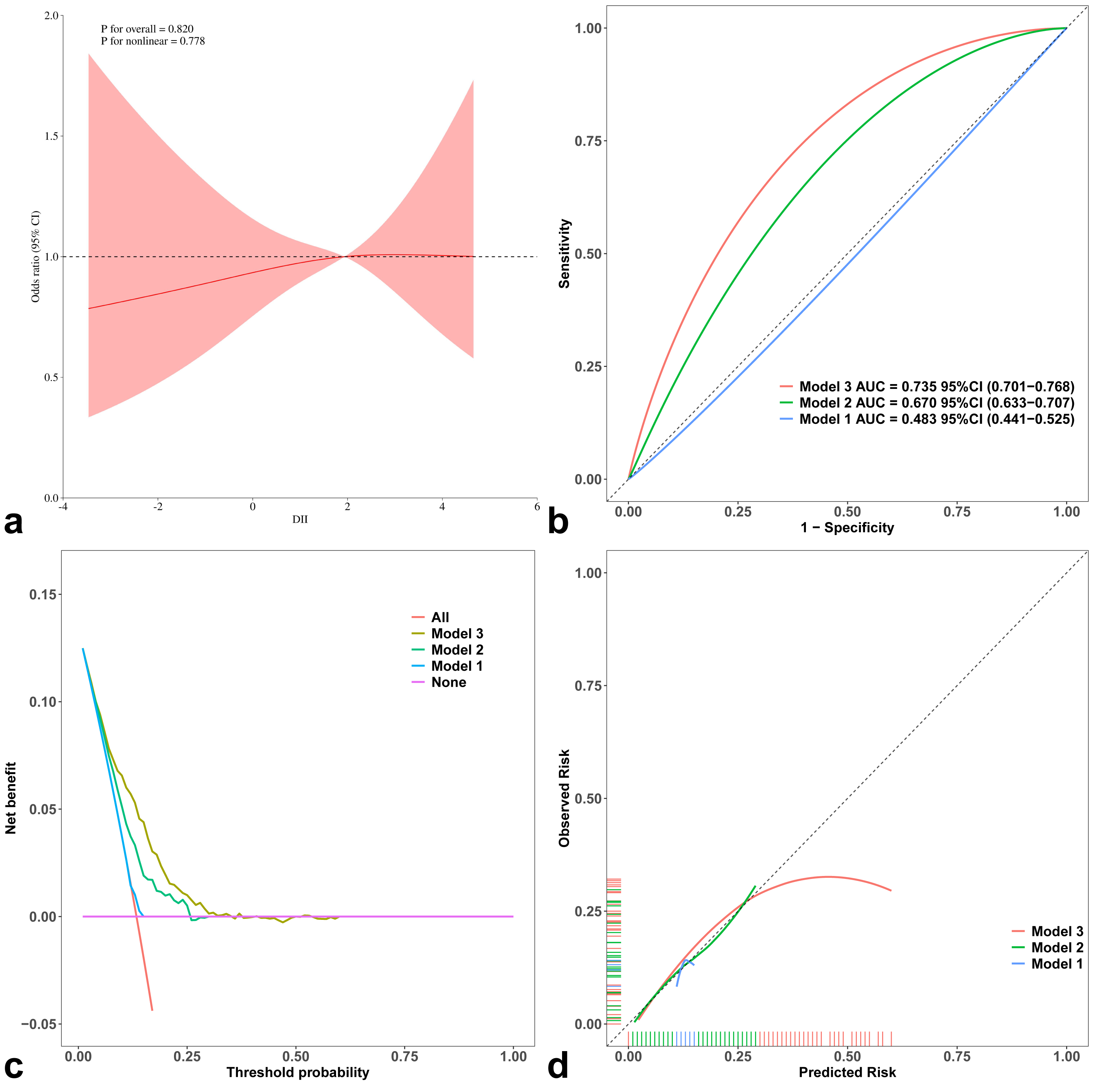
**

**Supplementary Figure 7. Performance evaluation of Multi-model incorporating confounders with RCS(a), ROC(b), DCA(c) and calibration curve(d) of CHD**

**
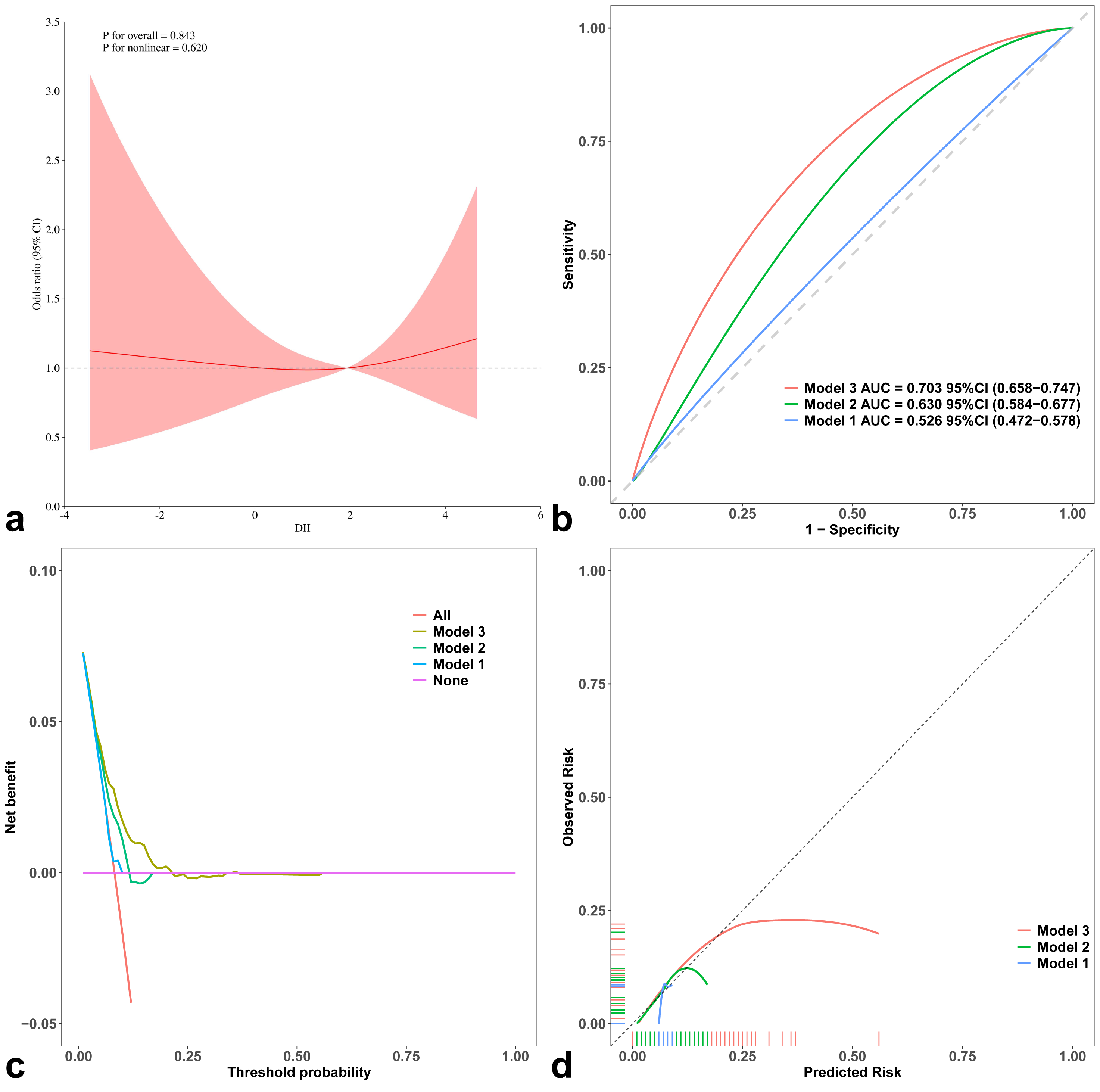
**

**Supplementary Figure 8. Performance evaluation of Multi-model incorporating confounders with RCS(a), ROC(b), DCA(c) and calibration curve(d) of Angina**

**
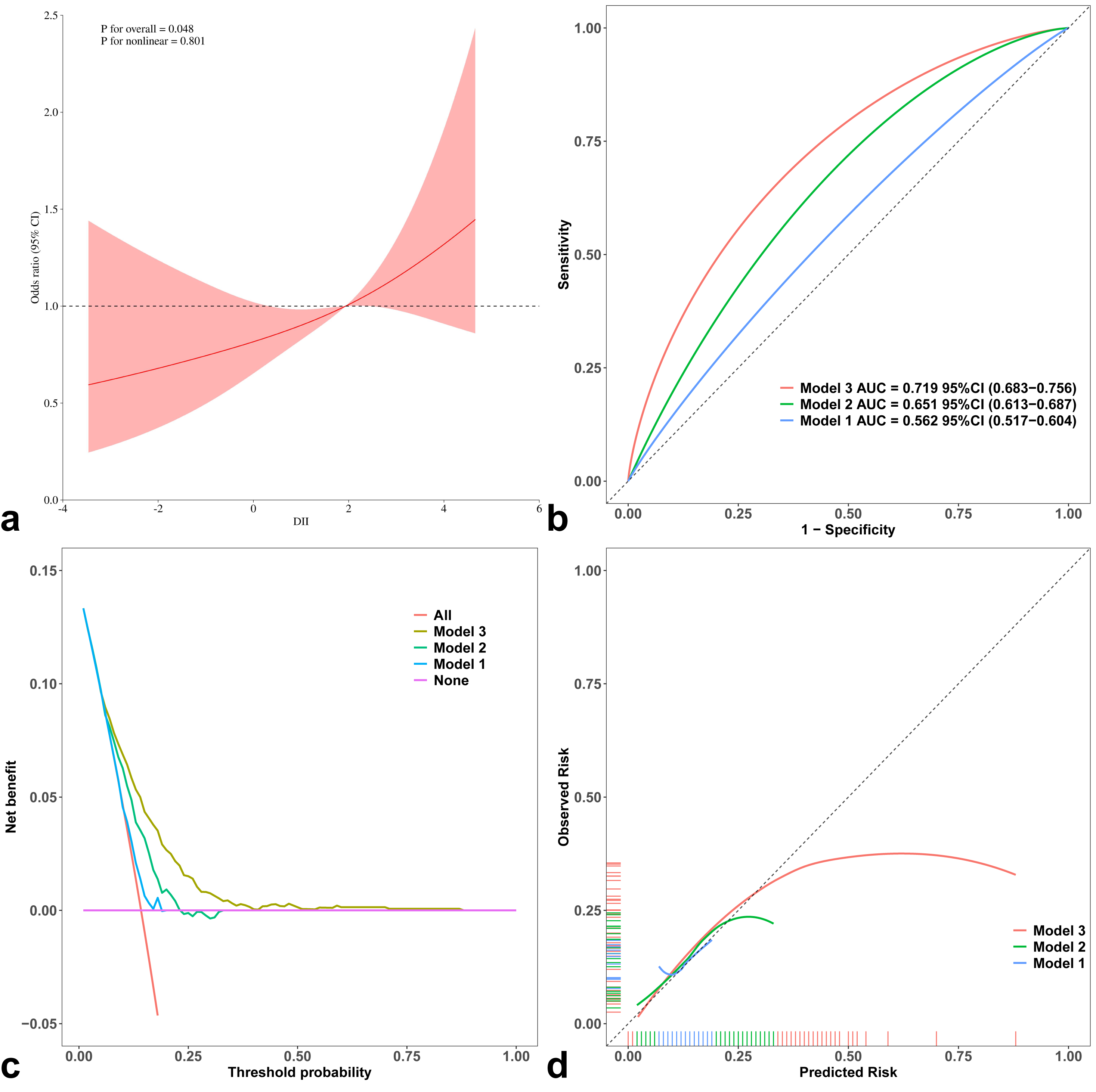
**

**Supplementary Figure 9. Performance evaluation of Multi-model incorporating confounders with RCS(a), ROC(b), DCA(c) and calibration curve(d) of MI**

**
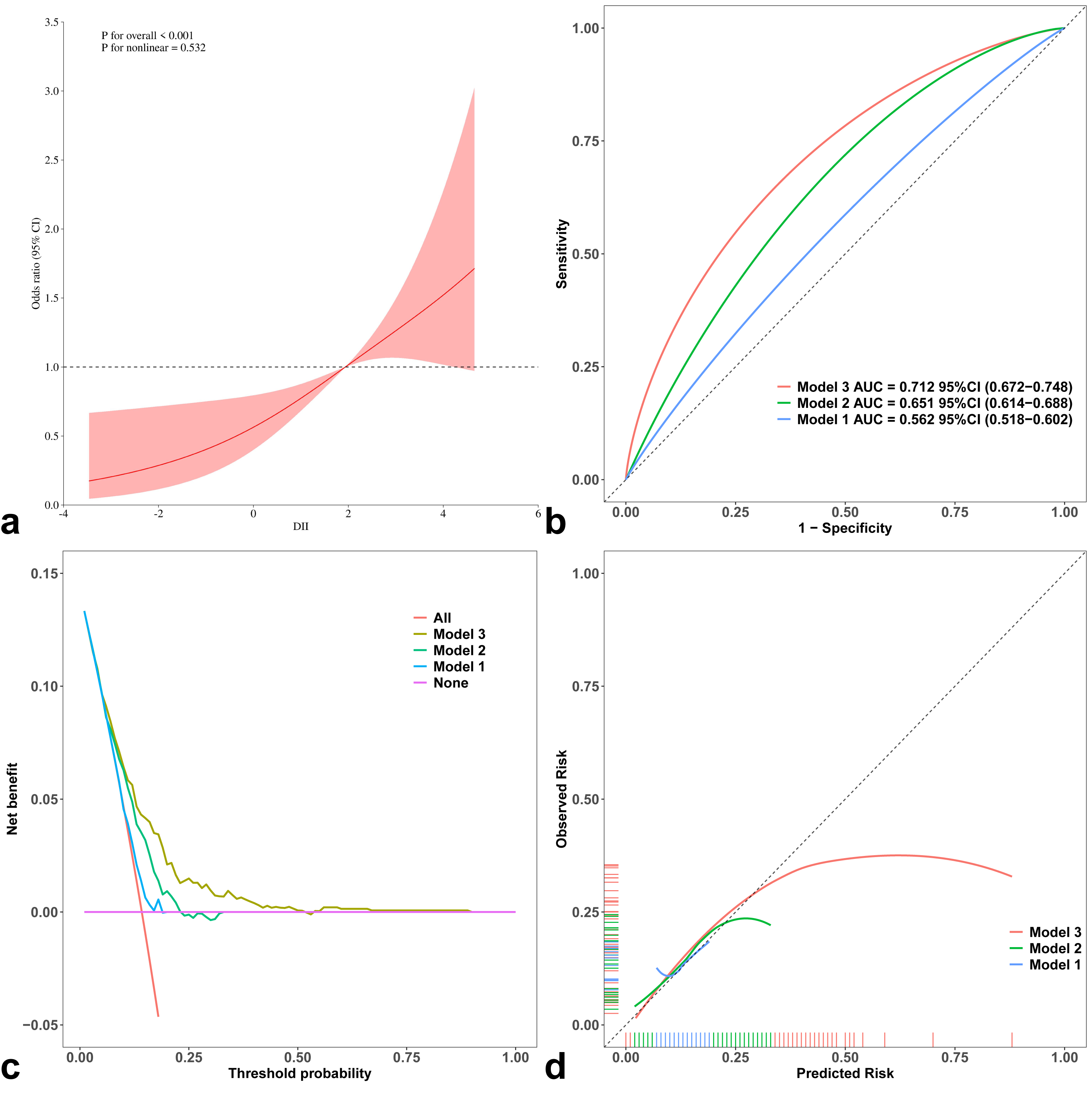
**

**Supplementary Figure 10. Performance evaluation of Multi-model incorporating confounders with RCS(a), ROC(b), DCA(c) and calibration curve(d) of Stroke**
